# Population genomic assessment of semi-captive Asian elephants (*Elephas maximus*) from Myanmar: endangered species management and conservation implications

**DOI:** 10.1186/s12864-026-12912-7

**Published:** 2026-05-09

**Authors:** Elisa Somenzi, Larissa S. Arantes, Ronan James O’Sullivan, Hansraj Gautam, Diogo J. Franco dos Santos, Zaw Min Oo, Win Htut, Camila J. Mazzoni, Virpi Lummaa

**Affiliations:** 1 Department of Biology, University of Turku, Turku, 20014 Finland; 2 Berlin Center for Genomics in Biodiversity Research (BeGenDiv), Königin-Luise-Straße 2-4, Berlin, 14195 Germany; 3 Department of Evolutionary Genetics, Leibniz Institute for Zoo and Wildlife Research (IZW), Alfred-Kowalke-Straße 17, Berlin, 10315 Germany; 4 Myanma Timber Enterprise, Yangon, 11011 Myanmar

**Keywords:** Asian Elephant, *Elephas maximus*, conservation genomics, Semi-captive populations, Myanmar

## Abstract

**Background:**

Genomic approaches can provide critical insights into the genetic health of endangered species and the impacts of long-term management on semi-captive populations. Asian elephants (*Elephas maximus*), listed as Endangered, include a large semi-captive population in Myanmar that may represent an important reservoir of genetic diversity. However, their genetic structure, levels of inbreeding, and relatedness remain poorly characterized.

**Results:**

We assembled the largest genomic dataset to date for semi-captive Asian elephants, comprising reduced representation data (RADseq, *N* = 261) and whole-genome data (WGS, *N* = 64). Heterozygosity values showed no significant differences between wild-born and captive-born individuals. Both RADseq and WGS data revealed low to medium levels of inbreeding and no evidence of an increase among younger generations. Population structure analyses confirmed a homogeneous population with no geographic-based genetic structure, likely reflecting management practices and natural mating with wild bulls. Demographic inference indicated a sharp decline in effective population size (*Ne*) between 60 and 30 generations ago, consistent with a long-term population contraction, and current *Ne* was estimated as being very low. Relatedness analyses identified 657 first-cousin or closer relationships, including 124 first-degree pairs. We also uncovered 35 previously undocumented father-offspring pairs with some males having disproportionately high reproductive success. To facilitate future monitoring, we developed three reduced relatedness-informative marker (RIM) panels. The smallest panel (274 SNPs) provided sufficient resolution for reliable parentage assignment at reduced cost.

**Conclusions:**

Our findings demonstrate how genomic tools uncovered the genetic consequences of management of the largest semi-captive elephant population of Myanmar, highlighting the need for continuous monitoring to safeguard its genetic diversity. More broadly, this study illustrates how integrating WGS and RADseq can inform conservation planning for semi-managed populations and offers transferable approaches applicable to other endangered species.

**Supplementary Information:**

The online version contains supplementary material available at 10.1186/s12864-026-12912-7.

## Background

Extinction rates are rising at an unprecedented pace, with 26% of mammal species assessed by IUCN Red List being threatened with extinction, and many others facing rapid decline in distribution and abundance [1]. These threats are especially pronounced for large-bodied animals due to hunting and habitat fragmentation [2]. Large herbivores, in particular, are both highly threatened and ecologically pivotal, as their decline disrupts key ecosystem processes [3]. Therefore, monitoring viability and conservation status of wildlife has become a main challenge for modern biology, with the need to provide reliable evidence to support management strategies and prevent further biodiversity loss [4, 5]. Traditional methods based on ecological demographic monitoring are a valuable tool in the assessment of endangered species viability but may not always accurately reflect the actual conservation status of a population. Genomic-based methods can provide detailed information on inbreeding levels, genetic diversity, and hybridization or introgression events [6–8]. Several cases have been documented of wildlife populations displaying high abundance, suggesting good conservation status, while further genomic assessments have revealed reduced genetic diversity, low numbers of genetically effective breeders, and a high incidence of deleterious mutations [8–11]. In the context of endangered species monitoring, genomic approaches have therefore emerged as powerful alternatives or complements to traditional methods [6]. For many species targeted genomic tools have been developed, such as low-density panels of genomic markers, to monitor populations in an efficient and cost-effective way [12–14]. Such tools and expertise are now imperative for effectively guiding conservation actions, including reintroduction, relocation, and the establishment of *ex situ* populations [15].

Asian Elephants (*Elephas maximus*) are deeply embedded in Asian history, culture, and religion and play unique ecological roles in many Asian countries [16]. Around 4000 BCE (Before the Common Era), the historical range of the species covered over 9 million km², but today this has been reduced to just 486,800 km² of highly fragmented habitats, with an estimated 48,323–51,680 individuals remaining in the wild and approximately 10,000 in captivity [16, 17]. These numbers have been declining for centuries as a consequence of hunting, illegal poaching, deforestation, agricultural expansion, and urbanization to the point that the species is currently listed as endangered on the IUCN Red List [18]. Despite their prominent role in Asian society and position as a flagship species for conservation, genetic diversity, levels of inbreeding, and population structure of the Asian elephant have only been studied at a coarse level [19, 20]. Modern, in-depth genomic assessments are scarce for Asian elephant populations located in India and almost completely absent from other Asian countries. Among the latter, Myanmar harbours the world’s second-largest wild population of Asian elephants and the largest semi-captive population of working elephants [21]. This semi-captive group comprises approximately 5000 individuals used primarily by the logging industry, 2700 of which are owned by the Myanma Timber Enterprise (MTE) [21]. Although subject to partial human management, this population reproduces naturally, with matings frequently involving wild conspecifics. Therefore, it is not genetically isolated from the wild. This unique and extensive population represents a potential reservoir of genetic diversity for this endangered elephant species, and it may serve in the future as an important source for reintroduction and relocation programs [22, 23]. Despite its relevance for the conservation of the Asian elephant, this population lacks a fine-scale assessment of its population structure, inbreeding, and genetic diversity.

In this study we analyse a genomic dataset of semi-captive working elephants, composed of whole genome sequencing (WGS) and Restriction site–Associated DNA sequencing (RADseq) data for 67 and 261 individuals, respectively. For each individual, information on birth or capture location, including exact or estimated birth date as well as maternal pedigree are available. We use these data to quantify genetic diversity and population structure of the semi-captive Asian elephant population of Myanmar, assessing the impact of human management and a free mating system on the levels of inbreeding at both geographic and temporal scales. Furthermore, we perform kinship analysis to detect father-offspring pairs, thus, enhancing the otherwise matrilineal pedigree available for this semi-captive population. Finally, we present newly developed low-density marker panels to confidently assess relatedness among Myanmar elephants.

Ensuring the survival of endangered species increasingly depends on integrating genomic tools into conservation practice [4, 6]. Our study provides, to our knowledge, the largest and most comprehensive genomic assessment of a population of Asian elephants, uncovering key patterns of diversity, inbreeding, and relatedness that are invisible to traditional monitoring. Beyond Asian elephants, this work aims to illustrate how the assessment of population structure and genomic diversity may contribute to conservation management, with potential implications for maintaining genetic health and long-term population viability.

## Materials and methods

### Study population

The Asian elephant population sampled for this study is used in the logging industry and the animals are property of the state-owned Myanma Timber Enterprise (MTE), which manages different working camps across the country [24]. These elephants work for around eight hours per day from the age of 18 until 55, when they are retired from the activities [25]. Their work consists of hauling and stacking logs, loading them for transport, and carrying materials or equipment across forested terrain. MTE elephants are marked in their haunches with a unique identifier, regularly health-checked by veterinarians, with maternity, births, and deaths recorded in log books. Elephants are sometimes relocated to different camps within Myanmar due to changing logging needs, with long-distance translocations also occurring. Outside of working hours, elephants are left free to roam in the forests around the camp, where they feed independently and rest until morning. Mating occurs naturally and unsupervised, both with semi-captive bulls and any wild bulls present in the area. Observing mating events is a rare occurrence, so the population’s pedigree is only complete on the matrilineal line. The study population includes animals born from MTE-owned elephants (captive-born) and individuals captured from wild herds (captive-born) [25]. In 1995, the Myanmar government declared any further wild-capture of elephants illegal [20]. Therefore, MTE now relies mostly on offspring from captive females for recruitment into the timber industry workforce [26].

### Sampling

**Fig. 1:**
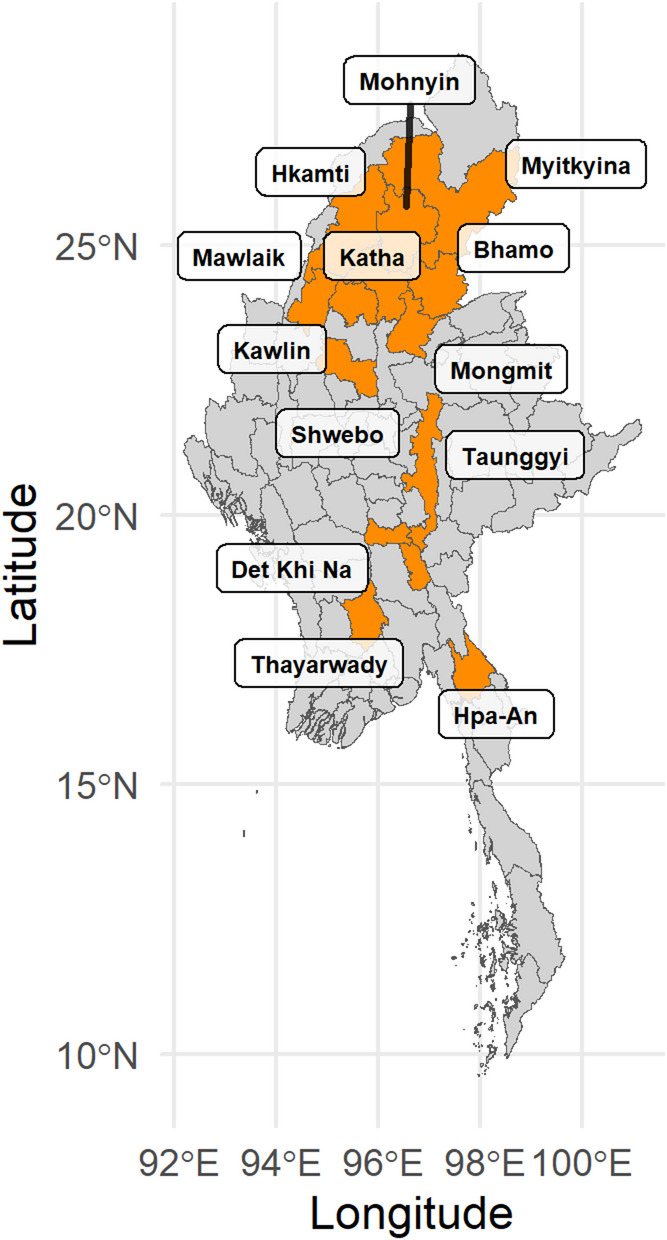
Map of Myanmar, districts highlighted in orange are the ones where elephants included in this study were born. Logging camp list and corresponding geographical district can be found in Supplementary Table 2

A total of 297 individuals were sampled, of which 261 were successfully genotyped (Supplementary Table 1). Blood was obtained from the vein behind the ear by authorized veterinarians as part of regular health monitoring, following both local and University of Turku ethical guidelines, and directly stored in ethanol. The elephant blood samples were imported from Myanmar in full compliance with the Convention on International Trade in Endangered Species of wild fauna and flora (CITES) regulations and in accordance with the principles of the Nagoya Protocol on access and benefit sharing. DNA was extracted using the DNeasy Blood and Tissue kit (Qiagen). The dataset consists of 158 females and 103 males, including both 54 wild-captured and 205 captive-born individuals, and two individuals for which the place-of-birth status was not available. The time range covered by the sampled individuals ranged from the oldest individual born in 1951 to the youngest born in 2015. All blood samples were collected at our study sites in West Katha, East Katha, and Kawlin between 2014 and 2018. Since many elephants had been transferred to these sites during their lifetimes, the dataset ultimately includes individuals born in 20 different logging camps across the country (Fig. 1). Consequently, the birth distribution across MTE camps is uneven: West Katha, East Katha, and Kawlin are represented by 38, 51, and 129 individuals, respectively, whereas the remaining camps are represented by only one to seven individuals each (Fig. 1; Supplementary Table 2).

### RADseq dataset

We produced Restriction site–Associated DNA sequencing (RADseq) data to investigate molecular diversity and population structure in our study samples. RADseq data provide a reduced-representation snapshot of the genome by sequencing DNA adjacent to restriction enzyme cut sites, allowing cost-effective genotyping of markers across many individuals. We followed the 3RADseq protocol [27], incorporating a modification from Hoffberg et al. [28] by adding an 8-base degenerate tag to the P5 adapter to allow identification and removal of PCR duplicates. Briefly, we used the restriction enzymes *EcoRI*, *XbaI*, and *NheI*, which enabled sequencing of approximately 23,500 loci ranging from 530 to 705 bp. The initial DNA input per sample was 200 ng. Each sample was digested with 10 U of each enzyme in NEB 1× CutSmart Buffer and ligated with 5 µM barcoded adapters. After a 2-hour incubation at 37 °C, DNA ligase and ATP were added, and the reaction was subjected to temperature cycling to promote ligation (22 °C) followed by digestion (37 °C), finishing with 80 °C for 20 min. Barcoded samples were pooled and subjected to fragment size selection using the Blue Pippin (Sage Science) with a 1.5% cassette, setting the selection range to 530–705 bp. The 40 µL size-selected product was split into four 10 µL aliquots, and each was subjected to a single-cycle PCR to incorporate the iTru5-8 N primer, followed by an indexing PCR (8–10 cycles) using P5 outer and P7 primers. Final libraries were assessed using an Agilent Tapestation with High Sensitivity DNA ScreenTape. A low coverage sequencing run (around 2,000 reads per individual) was conducted to screen samples for endogenous DNA content and assess the balance between individuals within each library. Based on the proportion of reads assigned to each individual, new volumes of the digestion/ligation product were calculated and re-pooled for a second library preparation to ensure equal representation across individuals. This weighted re-pooling strategy is described by Arantes et al. [29]. Final libraries were sequenced on an Illumina NovaSeq S4 platform using 150 bp paired-end reads, targeting ≥ 30× coverage per individual.

The raw RADseq data comprised 1,187,976,121 paired-end reads from 297 individuals. Sequences were demultiplexed, PCR duplicates removed, followed by the exclusion of short fragments less than 370 bp in length, reads containing restriction sites, and those failing a third-enzyme filter.

Preprocessed reads were mapped to the *Elephas maximus* genome (GenBank accession number GCF_024166365.1, [30]) using Bowtie2 with default parameters, along with the “--no-mixed” and “--no-discordant” options. SNP calling was performed using Stacks reference-based pipeline v2.61. Only loci genotyped in at least 60% of individuals in at least one population (*p* = 1, *r* = 0.6) were retained (68,849 SNPs).

For SNP selection, a Phred score threshold of 30, minimum coverage threshold of 10 and a maximum of 100 were applied, alongside a minor allele frequency (MAF) cut-off of 0.05 (23,486 SNPs). To minimise linkage disequilibrium, only one SNP per locus was retained. SNPs with more than 10% missing data were excluded.

### Whole genome sequence (WGS) dataset

Aiming at producing a high-resolution dataset to validate trends highlighted by RADseq data, a subset of 64 individuals, evenly distributed between wild- and captive-born, was selected from the full set of 297 individuals and whole genome sequenced. Whole-genome libraries were prepared using the NEBNext Ultra II FS DNA Library Prep Kit (Illumina). Approximately 150 ng of total genomic DNA was enzymatically fragmented for 8 min. Adapter ligation was performed, followed by size selection of fragments between 500 and 700 bp using CleanPCR magnetic beads (GC Biotech). Libraries were then amplified with four PCR cycles using i5 and i7 NEBNext Multiplex Oligos (Illumina). Final libraries were sequenced on the Illumina NovaSeq S4 platform with a 300-cycle paired-end kit. Adapter removal was done using Trimmomatic v0.39 [31] and sequences aligned to the reference Asian elephant genome (GenBank accession number GCF_024166365.1, [30]) using bwa mem2 algorithm as implemented in BWA v0.7.19 [32]. Aligned sequences were sorted with SAMtools v1.22 [33] and duplicates marked using Picard *MarkDuplicates* v3.1.1 [34]. Quality filtering was performed on each sequence with SAMtools v1.22; reads with a mapping quality score below 40 were removed, and only paired and mapped reads were retained. Variant calling was conducted using GATK v4.5.0.0 [35], with bases whose Phred score was below 30 and whose sequencing depth was below one-third mean coverage or above 2X mean coverage filtered out. Sites located on sex chromosomes were excluded to avoid the introduction of bias in analyses designed for autosomal data. PLINK v1.9 [36] was used to retain only biallelic variants with no missing values and a MAF > 0.05. Loci in linkage disequilibrium were identified and removed using the ‘--indep-pairwise’ function in PLINK, where SNPs with r^2^ > 0.25 were removed from sliding windows of 50 kb and a step size of five SNPs.

### Genetic diversity and inbreeding level

Observed heterozygosity (*Ho*) values were calculated independently for each individual with the RADseq dataset using PLINK v1.9. Inbreeding coefficient *F*_*ROH*_ for each individual was calculated as the proportion of the genome covered by runs of homozygosity (ROHs). ROHs were assessed separately for each individual with PLINK v1.9. As described by Khan and colleagues [18], a ROH was defined if the following criteria were met: (i) absence of heterozygous genotypes, (ii) less than two missing genotypes, (iii) a minimum of 20 SNPs per ROH, (iv) a minimum SNP density, calculated as the ratio between the number of SNPs in the dataset and the total genome length, and (v) a minimum ROH length of 100 kb. RADseq data, often characterized by a low-density of markers and high levels of missingness, are known to underestimate the proportion of the genome covered by homozygous segments. Consequently, ROHs were also assessed on the subset of individuals for whom WGS data were available. Again, the analyses were performed using PLINK v1.9 with parameters from previous work on WGS elephant data [19]. After testing the assumptions of normality, group differences in *F*_*ROH*_ (i.e., genome fraction in ROHs) and *Ho* between captive and wild individuals were evaluated using a Wilcoxon rank-sum test in *R*. Individuals were grouped according to decade of birth, and differences in inbreeding among decades were tested using ANOVA in *R*.

### Population structure analyses

Prior to population structure analyses, the RADseq dataset was filtered to exclude closely related individuals to avoid biases in population structure analyses, as their presence can inflate signals of genetic similarity and hide population-level patterns. A KING-relatedness cut-off of 0.08 was applied using PLINK v2.0 to remove individuals that were 2nd degree, or closer, relatives [37]. This threshold was applied uniformly across all camps and sampling years. One individual from each kin pair was removed, prioritising geographic distribution and camp representativeness. To investigate whether individuals clustered according to their camp of origin, samples were labelled based on their respective camp.

Principal Component Analysis (PCA) and admixture analysis were then performed on both the complete dataset and a subset consisting exclusively of wild-captured individuals. PCA was carried out using PLINK v1.9 and results visualised in R v4.4.1 with *ggplot2* [38].

Unsupervised clustering was performed using ADMIXTURE v1.3.0 [39] for values of *K* ranging from 2 to 10, and the optimal number of clusters was determined using five-fold cross-validation (CV) errors. Results were visualised using *BITE* v2.0 [40].

To investigate the recent and historic demographic history of the population, we used the WGS and RADseq datasets to investigate the length and distribution of Identity-by-Descent (IBD) segments. A Hidden Markov Model (HMM) was applied to differentiate between IBD and non-IBD segments across the genome using *RZooRoH* 0.4.1 [41]. This approach allowed us to differentiate classes of IBD according to segment length: this allows us to distinguish recent inbreeding from founder effects or past population bottlenecks. IBD segments were grouped into nine length-based classes, each corresponding approximately to shared ancestry from 2, 4, 8, 16, 32, 64, 128, 256, and 512 generations ago, along with an additional class representing non-IBD segments.

The contemporary effective population size (*Ne*) was estimated using CurrentNe v1.0.0 [42], which employs artificial neural networks to model the relationship between linkage disequilibrium patterns and *Ne*. Analyses were conducted on the full set of individuals, as sample size has a more substantial impact on *Ne* estimation accuracy than the number of markers when the latter exceeds 1000 [42]. Confidence intervals at the 50% and 90% levels were calculated to assess the precision of *Ne* estimates.

Historical trends in *Ne* were estimated using both WGS and RADseq datasets using the LD-based method implemented in GONe v2.0 [43]. A constant recombination rate of 1 cM (centimorgan) per megabase pair was assumed, as recommended for mammals [44]. To mitigate potential biases arising from population substructure in our estimates of more recent historic demography, we considered only pairs of loci within 2 cM. We included 50,000 single nucleotide polymorphisms (SNPs) per chromosome, as the maximum amount allowed by the software, and repeated the analysis 100 times, each with a different randomly selected set of SNPs per chromosome. Repetitions were used to calculate confidence intervals for historic *Ne* estimates.

### Relatedness

Relatedness between each pair of individuals in the RADseq dataset was assessed using KING-robust kinship estimator [37], as implemented in PLINK v2.0. Relatedness results were used to cross-check pedigree assignments and to identify unknown fathers.

To identify a reduced number of relatedness informative markers (RIM), we filtered the RADseq dataset following the pipeline described in Andrews et al., 2018 [45]. The post-quality control dataset underwent a more stringent filtering, where markers with a call rate below 5% and a linkage disequilibrium *r*^*2*^ value above 0.2 were removed using PLINKv1.9. We then selected high-frequency SNPs since such markers are generally quite informative of population structure due to their greater heterozygosity. We applied three different MAF thresholds (0.35, 0.40, 0.45) to create three RIM panels with different numbers of markers.

To test the accuracy of our RIM panels in assessing relatedness, we first calculated KING kinship coefficients for each pair from the full set of 13,030 SNPs using PLINK v2.0. We then recalculated kinship coefficients using each RIM panel. Pearson correlation values between kinship coefficients obtained from each of the four panels was assessed in R with the cor() function. Finally, we assessed if the SNPs identified were in fact more informative than the rest of the available markers. We randomly selected the same number of markers from the full set of SNPs as the RIM panels and tested the correlation between kinship coefficients obtained from the reference set and from these random panels. We iterated over this process 100 times and the mean correlation value obtained was compared with the correlation value obtained from the RIM panels.

## Results

### Working datasets description

After quality control and pruning, the RADseq dataset comprised 13,030 SNPs and 261 individuals. The WGS subset of individuals yielded a post-QC dataset of 3,890,189 SNPs across 64 individuals, with a mean post-QC coverage ranging from a minimum of 10.6x to a maximum of 23.9x. This subset included 29 males and 35 females, with 32 individuals captured from the wild and 32 born to MTE-owned mothers. In this reduced dataset, 15 MTE camps out of 20 are represented.

### Genetic diversity and inbreeding

We found no evidence that measures of genetic diversity and inbreeding in the population would have changed in the most recent decades, or that the captive-born elephants would have presented lower diversity or higher inbreeding values as compared to those captured from the wild.

First, values of heterozygosity (*Ho*) ranged from a minimum of 0.15 recorded for a captive-born female from Kawlin born in 1974 to a maximum of 0.29 recorded for a wild-born female captured near the same location, born ~ 1968. Mean *Ho* for females was slightly higher than for males, 0.190 and 0.187, respectively (*p-value* = 0.018). There was no significant difference in mean *Ho* between wild-born (mean = 0.185) and captive-born (mean = 0.189) elephants (*p-value* = 0.061) nor between decades of birth (min: 0.182–1950 s and max: 0.191–1980 s, *p-value* = 0.52).

Second, inbreeding values calculated using RADseq data ranged from 0.01 for a wild-born female born ~ 1968 to a maximum of 0.24 recorded for a captive-born female born in 1974. The mean inbreeding value for captive-born individuals was 0.044 and 0.045 for wild-born individuals, with no significant difference (*p-value* = 0.0739). Inbreeding values obtained from WGS data were higher, ranging from 0.07 to 0.32. Inbreeding estimates from RADseq data were on average 9% less than those obtained from WGS. However, the overall trend was consistent among individuals, with the most inbred individuals scoring the highest values independent of marker density (Supplementary Fig. 1). The correlation value between F_ROH_ results obtained with WGS and those obtained with RADseq was 0.78.

The three camps with highest sample representativeness scored comparable values of average *Ho* and inbreeding: 0.182 and 0.045 for East Katha, 0.190 and 0.045 for West Katha, and 0.19 and 0.044 for Kawlin (Supplementary Table 2). There was no difference in mean inbreeding values for individuals grouped by decades of birth (*p-value* = 0.466), suggesting that there has been no change in inbreeding since the mid-20th century among the MTE elephant population (Supplementary Fig. 2).

**Fig. 2:**
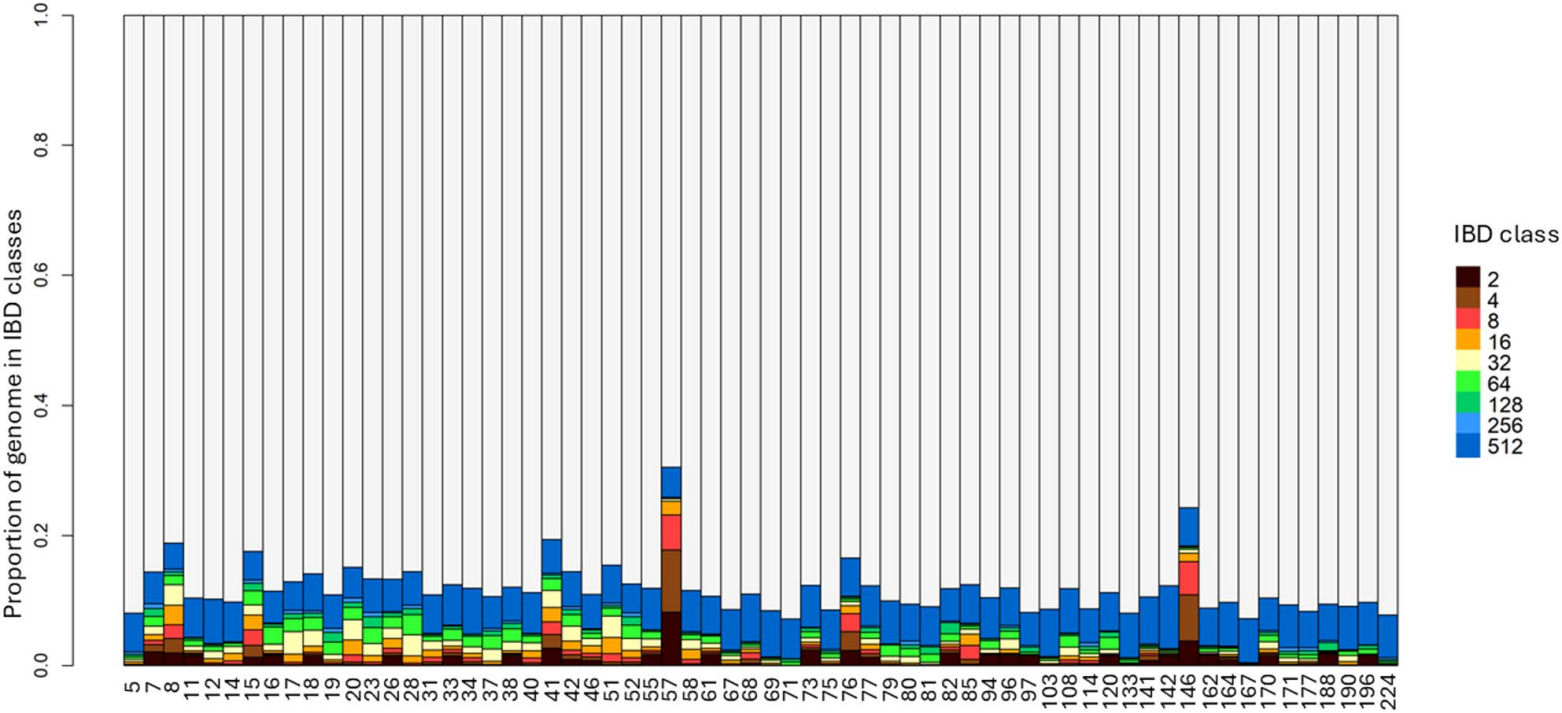
Partitioning of Identity-by-Descent (IBD) segments across individuals. Bars represent the proportion of the genome assigned to different IBD classes with colour code representing a shared ancestry from 2, 4, 8, 16, 32, 64, 128, 256, and 512 generations ago

To investigate patterns of recent and ancient relatedness within the population, IBD segments were grouped into nine length-based classes, corresponding to approximate shared ancestry from 2 to 512 generations ago. In results obtained from the WGS data, most individuals exhibited a predominance of older IBD classes, indicating low levels of recent inbreeding across the population (Fig. 2). This pattern suggests that the majority of shared ancestry derives from more ancient common ancestors. However, a subset of individuals displayed noticeable contributions from more recent IBD classes (e.g., 2, 4, 8), reflecting the presence of sporadic inbreeding events. RADseq data presented a higher occurrence of IBD segments from medium to old classes (Supplementary Fig. 3). This discrepancy between patterns observed with WGS and RADseq data is most likely due to the lack of fine scale definition and uneven distribution of markers across the genome that characterises low-density data.

### Population structure

**Fig. 3:**
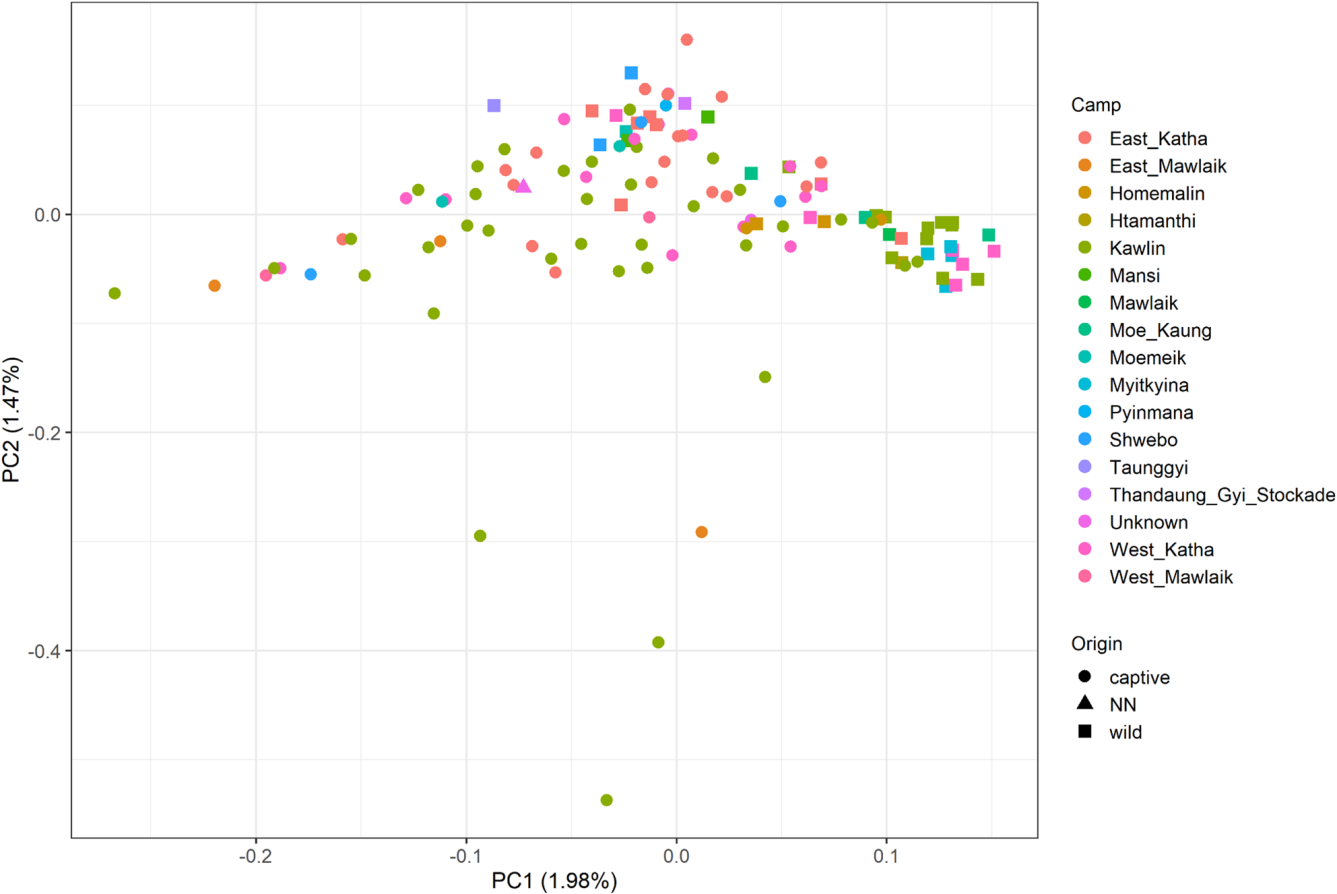
PC1 vs. PC2 of the unrelated sample set. Colour of the points is according to camp of origin. Shape is according to the wild or captive origin

Next, we investigated if genetic variation was correlated to the geographic distribution of the elephant birth/capture locations across different MTE camps in Myanmar but found no support for this among either captive-born nor wild-born individuals. The first principal component (PC1), calculated from the unrelated subset of individuals, explained 1.98% of the total genetic variance (Fig. 3). PC1 revealed a group of wild-born individuals in the upper right quadrant of the plot. More than half of the wild-captured individuals grouped within this cluster, despite having been captured near different camps. This clustering trend of wild-captured individuals was however not confirmed by any further analysis. The second principal component (PC2), accounting for 1.47% of the variance, highlighted several outlier individuals, most of whom were captive-born and originated from the Kawlin camp, with the exception of one sample from East Mawlaik (Fig. 3).

Admixture analysis did not reveal any strong signal of camp-based clustering (Supplementary Fig. 4). One possible explanation for the absence of such structure is the presence of captive-born individuals. To investigate population structure without the influence of captivity, a further Admixture analysis was performed using only wild-captured individuals. The results from this subset also showed no evidence of geographic (capture camp based) clustering (Supplementary Fig. 5). The optimal number of clusters, as evaluated via CV errors, was identified as *K* = 7 for the analysis on all the individuals and *K* = 2 for the subset of wild animals only (Supplementary Fig. 6a and 6b). However, given the absence of clear population structure, these inferred clusters likely reflect subtle allele frequency differences rather than discrete populations.

### Demography

**Fig. 4:**
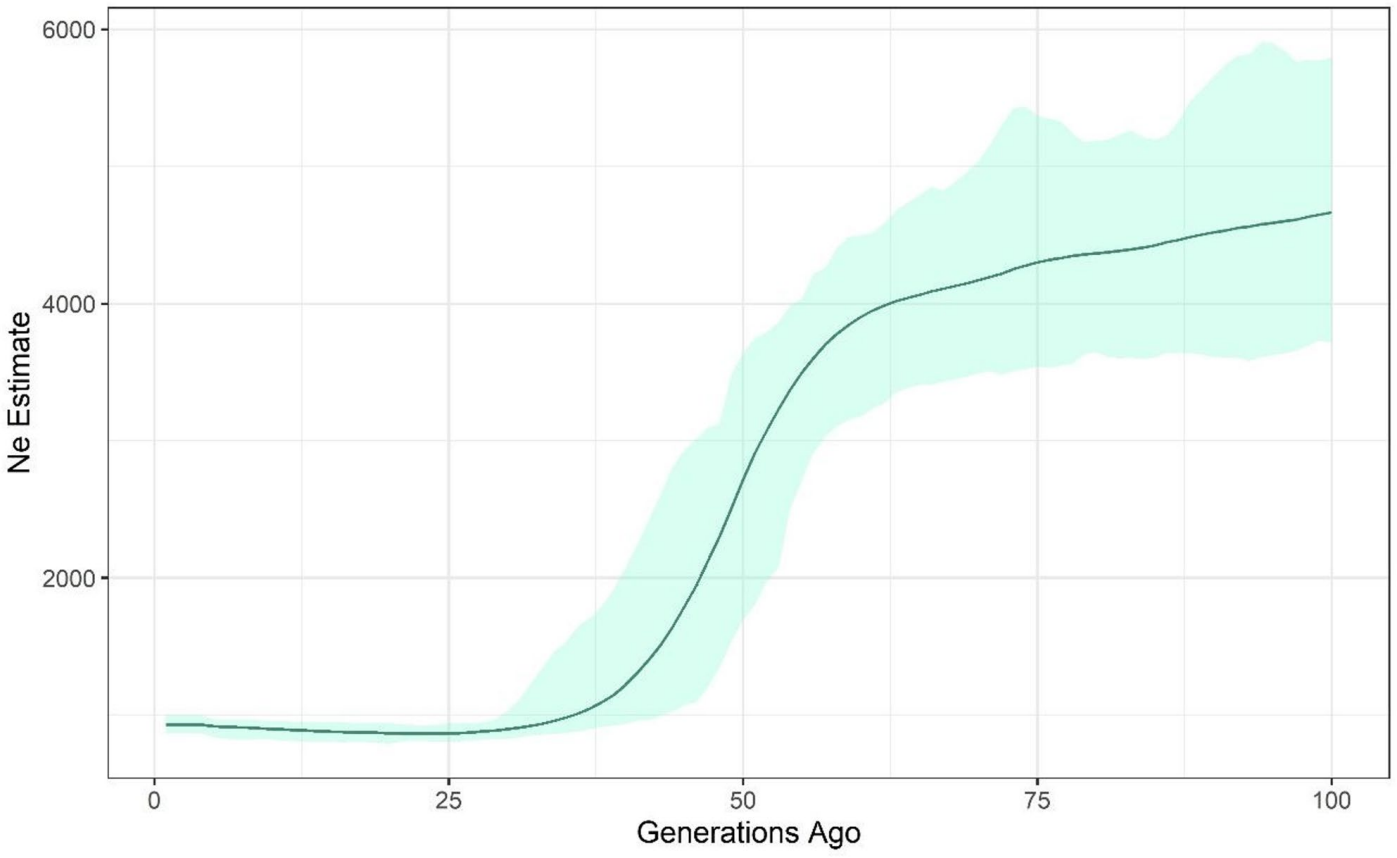
Historical estimates of effective population size (Ne) over the past 100 generations, inferred using the GONe software over 100 replicates on the WGS dataset. The solid line represents the median Ne estimate, while the shaded area indicates 95% confidence intervals

To investigate long-term trends in effective population size (*Ne*) and assess potential changes over time, we conducted demographic analyses using both RADseq and WGS datasets. Based on the CurrentNe software analysis which used the RADseq dataset, contemporary *Ne* was 149.55 individuals (90% CI: 141.97-157.54). Historical demographic trends assessed with GONe on the WGS dataset suggested that approximately 100 generations ago, the estimated *Ne* was relatively high (mean *Ne* based on 100 iterations: 4664 individuals). A steady decline in *Ne* is observed, particularly pronounced between 60 and 30 generations ago, indicating a likely demographic contraction (Fig. 4). From roughly 30 generations ago to the present, *Ne* stabilises at a relatively low level (less than 1000 individuals), suggesting a prolonged period of small recent *Ne*. When performed using the RADseq data, the same general pattern of demographic decline was observed. In addition, a very recent sharp decline (5 generations ago) was observed in the RADseq-based results but was not present in the WGS-based results (Supplementary Fig. 7). However, it cannot be excluded that this trend represents an artefact introduced by RADseq data, characterised by a lower marker density and higher missingness when compared to WGS data.

### Relatedness

To build a more complete pedigree of the semi-captive elephant population in the MTE camps, we used the RADseq dataset to assign parentage to the genotyped individuals. We identified 657 pairs of fourth-degree relatives e.g. first cousins (kinship coefficient between 0.125 and 0.25), 321 pairs of second-degree relatives e.g. half-sibs, avuncular, grandparental (kinship coefficient between 0.25 and 0.45), and 124 first-degree relatives e.g. parent-offspring, full-sibs (kinship coefficient above 0.45). We were able to identify 35 previously unknown pairs of father-offspring relationships, namely, a first-degree relationship where the older individual was male, the age difference between individuals exceeded 18 years, and no other relatedness (e.g.: same mother) between them was recorded in the pedigree. Specifically, we identified two particularly prolific bulls: one that sired nine elephants in our dataset, and another that sired six. Both of these bull elephants were already old animals when captured from the wild.

**Fig. 5:**
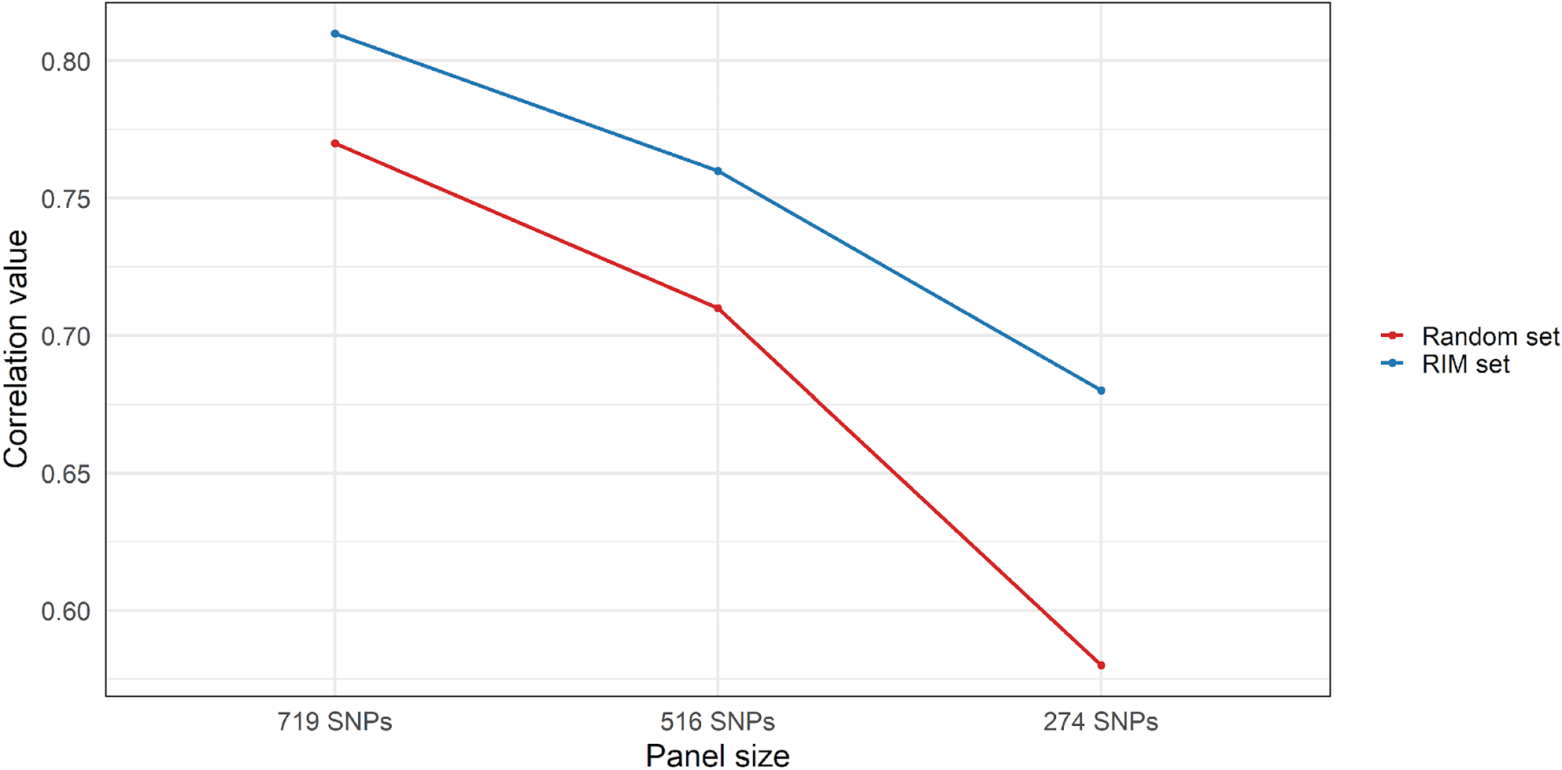
Correlation values between kinship coefficients estimated using the full SNP dataset and those obtained from reduced panels of relatedness-informative markers (RIM set – blue line) and random SNP panels of equivalent sizes (Random set – red line)

We selected a small number of relatedness informative markers in order to enable cost-effective pedigree inference, aiming at providing a valuable tool for parentage monitoring in wild and semi-captive Asian elephants. After filtering and applying three different MAF thresholds (0.35, 0.40, and 0.45), three RIM panels comprising 791, 516, and 274 SNPs, respectively, were obtained. SNPs in each of our RIM panels were evenly distributed across chromosomes in proportion to chromosome size (Supplementary Table 3). The correlation coefficients between kinship estimates derived from the full SNP set and each of the reduced panels were 0.81, 0.76, and 0.68, respectively (Fig. 5 – blue line). In comparison, the mean correlation coefficients from 100 replicates of random SNP panels of equal sizes were 0.77, 0.71, and 0.58 for the 791, 516, and 274 SNP panels, respectively (Fig. 5 – red line).

## Discussion

We present and analyse the biggest available genomic dataset of semi-captive Asian elephants to determine the conservation status and impact of human management on the Myanmar population. We found low to medium levels of inbreeding, with no evidence of an increase in inbreeding among younger generations, and a lack of geographically-based genetic structure, most likely caused by the constant gene flow with wild populations. Based on coalescent modelling, *Ne* is low with a marked period of demographic decline starting between 60 and 30 generations ago. Finally, we used our relatedness analysis to enhance an existing, exclusively matrilineal pedigree and proposed a set of markers for cost-efficient relatedness assessment of Asian elephants.

### Genetic diversity and inbreeding

The average values of inbreeding per decade of birth were consistent over time, with no differences in inbreeding levels between wild vs. captive-born individuals, or between samples from different geographic areas. Inbreeding levels estimated from WGS data were comparable to those reported for wild elephant populations in India [19]. In Myanmar, semi-captive elephants are typically allowed to roam freely around the camps at night, creating opportunities for interactions and mating with their wild counterparts, as previously reported by other authors [20, 24, 25]. This suggests that, despite being a managed and tamed population, mating may occur without human supervision with females often choosing to mate with wild bulls. Although widely reported, this occurrence has not yet been demonstrated from a genomic perspective, as no parent–offspring triplets involving MTE-owned elephants and wild individuals have been genotyped. However, two alternative explanations are also plausible. First, it is not uncommon for elephants to be transferred between camps by the MTE team, which could facilitate long-distance gene flow. Second, the relatively short duration of semi-captivity – encompassing only a few generations – may have been insufficient for the accumulation of significant unique genetic variation or substantial changes in allele frequencies. Considering that our findings may indicate ongoing reproductive connections between semi-captive and wild populations, gene flow coming from wild elephants could play an important role in maintaining genetic diversity within working elephant populations. Such genetic exchange may help counteract the potential negative effects of inbreeding that could arise if only a limited portion of the semi-captive population contributes to reproduction. However, given that our dataset captures only three generations of elephants in semi-captive conditions and Asian elephants have a long generation time, the full impact of current management practices on inbreeding and population structure may become more apparent over the long term.

It has already been demonstrated that Asian elephants do not breed easily in captivity, especially in contexts where groups are structured artificially [46–48]. Birth rates in MTE elephants have been found to be intermediate, falling between the minimum values observed in fully captive zoo elephants and the maximum values recorded in wild elephants [24, 48]. In wild conditions, Asian elephants live in matriarchal groups only composed of related females and their offspring, with males leaving the maternal herd upon reaching sexual maturity [16]. This behaviour, along with the long-ranging nature of the species, greatly reduces the risk of consanguinity. Furthermore, elephants in nature are known to have developed behavioural inbreeding avoidance and rely on a sophisticated chemosensory system for individual recognition, thus, reducing the risk of close-kin matings [49, 50]. Three individuals in our study possessed long IBDs and/or high inbreeding values which demonstrates that despite the population being outbred, sporadic inbreeding does occur. Although these elephants represent a minority within the sampled population, it is important to consider that semi-captive conditions disrupt natural herd structure and potentially limit mate choice. The two most inbred samples were a mother and her son, highlighting how pivotal it is to rely on genomic data to assess relatedness and support population management strategies. To accurately assess these patterns of relatedness and inbreeding across populations, we relied on RADseq sequencing, which is a valuable approach for capturing genetic diversity of several individuals in a cost-effective way, especially for those species for which commercial SNP arrays are not available. However, this approach presents some limitations: it has been demonstrated that RADseq data are more prone to allelic dropout, uneven coverage across loci and higher occurrence of SNP calling errors [51, 52]. These biases can deflate inbreeding estimates [53, 54] underestimate genetic diversity or fail to identify loci under selection in adaptation studies [52, 55]. To address this limitation, we applied stringent filtering parameters to the RADseq data and complemented RADseq-based analyses with WGS-based ones. In our study, results obtained from WGS and RADseq data were largely concordant. However, given that the RADseq dataset represents a relatively low-density SNP panel (fewer than 20,000 SNPs), we relied on WGS-based results in cases of discordance, particularly for analyses requiring fine-scale resolution. The combined use of RADseq and WGS data s allowed us to cross-validate inbreeding estimates and identify signals of recent inbreeding with higher resolution.

### Population structure and Ne

MTE elephants do not show geographically-based genetic clustering, appearing as a homogeneous population with high levels of genetic diversity. No genetic differentiation was detected between captive- and wild-born individuals, with the latter considered representative of the wild population at the time of their offtake, as capture practices historically targeted free-ranging herds [20]. These results are in line with those of Maurer et al. [56] who analysed microsatellite data for semi-captive elephants from Myanmar and Laos. They reported genetically homogeneous populations with almost no geographical differentiation and high levels of genetic diversity. We confirmed that, despite our samples covering a large geographical extent and distinct landscapes in Myanmar, these semi-captive elephants from Myanmar form a single homogeneous population with no sub-clustering. This lack of geographic genetic structure may be explained by two factors: [1] ongoing mating with wild bulls, which facilitates gene flow between wild and semi-captive populations; and [2] human-mediated movement of elephants between camps by the MTE, promoting long-distance gene flow. Together, these processes likely contribute to the long-term genetic health and homogeneity of the population. The absence of clear genetic structure among semi-captive individuals does not by itself demonstrate ongoing connectivity with wild populations, but it suggests that such gene flow is possible and consistent with the observed patterns. Direct evidence of exchange between wild and semi-captive populations would require more explicit sampling including wild individuals. Due to logistic constraints related to sampling, camps located in the southernmost part of the country are underrepresented in our study, with more than 80% of individuals being sampled in the three main camps, located in the central-north part of Myanmar. Although our analyses suggest a lack of genetic differences between northern and southern samples, we cannot a priori exclude the possibility that part of the existing genetic diversity was not captured by our sampling.

Semi-captive populations can serve as an important reservoir of genetic variation for endangered species, helping to maintain molecular diversity and, through their spatial distribution, may facilitate gene flow between isolated wild herds. This gene flow can counteract the negative effects of habitat fragmentation and inbreeding, potentially leading to genetic rescue in small, declining wild populations [57]. However, the conservation value of these semi-captive populations relies on the preservation of the ongoing gene flow, along with inbreeding monitoring, and limiting disproportionate reproductive success among individuals. Furthermore, captive populations are subject to various challenges, including elevated mortality rates [25]. These factors pose a threat to population survival, also potentially impacting *Ne*. Our results highlighted low contemporary *Ne*, with several possible factors that may have caused it. First, historical bottlenecks associated with the extensive past use of elephants for military and work purposes could have affected long-term genetic diversity. Second, the presence of highly prolific bulls may have increased reproductive skew, limiting the number of males effectively contributing to the next generation. Finally, our sampling scheme do not fully capture the contribution of wild individuals, particularly given that semi-captive and wild populations are not genetically isolated. Analysis of IBD segments identified a high frequency of short runs of homozygosity, suggesting a common shared ancestry in the distant past, supporting the occurrence of prolonged bottlenecks. These results are consistent with the Asian elephant’s history of constant exploitation since antiquity for use in armies and as working animals [16]. According to our results, *Ne* trends for recent generations are no longer on a steep decline, but they remain consistently low. Despite numerous conservation efforts, there are still no detectable signs of population size recovery, perhaps because nearly 70% of the global Asian elephant population occurs outside protected areas [18]. A low *Ne* is particularly concerning for long-lived species such as the Asian elephant, where long generation times slow genetic and demographic recovery, even under favourable conditions. Consequently, stable or increasing census numbers may create a misleading perception of demographic security, while a limited number of breeding individuals can still constrain genetic diversity and increase long-term extinction risk.

### Relatedness

In this study, we reconstructed an enhanced pedigree for the semi-captive Myanmar elephant population, enabling more detailed investigations of genetic and social structure. The identification of previously unresolved relationships, particularly paternal links, provides a foundation for future research into male reproductive success, female mate choice, and the influence of kinship on social organization [58–60]. Relatedness assessment enabled the identification of highly prolific bulls within the population, a particularly important finding for conservation efforts, especially in endangered populations. In Asian elephant society, it has been shown that older, larger males typically obtain more mating opportunities than younger individuals [61]. The presence of these older, highly prolific males may lead to reproductive skew, particularly in small, confined populations. This imbalance can have deleterious consequences, increasing the discrepancy between the census population size and the effective population size. The effective population size may decline disproportionately when only a few males sire a high proportion of the offspring, potentially reducing genetic diversity and increasing the risk of inbreeding and founder effects. Therefore, the monitoring of highly prolific males should be considered a key component of conservation strategies, as it helps safeguarding long-term population viability. Genomic relatedness assessments also confirmed matrilineal pedigrees, underscoring the accuracy of MTE veterinary record-keeping over more than a century. To further refine the pedigree and improve its utility for behavioural, demographic, and evolutionary analyses, it is essential to expand genotyping efforts to a larger proportion of the population. Achieving this goal will require the development and implementation of more cost-effective genotyping strategies.

Relatedness estimation can be effectively achieved with a moderate number of markers, provided that the markers are informative and appropriately selected [62–64]. Following criteria applied by Andrews et al. [45], we were able to identify panels of informative markers that on average performed around 10% more effectively than a panel of the same size of random markers. As expected, reducing the panel size was associated with a decrease in accuracy of kinship assessment. However, we demonstrated that a carefully selected subset of 500 to 1,000 SNPs can still estimate relatedness with over 75% correlation compared to assessments using several thousand of markers. Notably, the 274-SNP panel, despite its reduced size, provided sufficient resolution for reliable genotyping and parentage assignment, making it a practical and cost-effective tool for future research. The accuracy of these panels depends on how well the reference population captures the genetic diversity of the broader population, and in systems with large population sizes or low genetic variation, more markers may be needed. Our SNP panel development was based on RADseq, a cost-effective method well suited for conservation genomics in non-model species. Once validated, these reduced panels can support ongoing genetic monitoring of relatedness and inbreeding across generations, contributing to effective long-term genetic management of semi-captive populations.

## Conclusions

The dataset analysed in this study is the most extensive collection of genomic data on semi-captive Asian elephants curated to date. We present a detailed picture of the genetic make-up of Myanmar timber elephants, confirming previous findings on population homogeneity throughout the country, as well as inbreeding levels comparable with those reported for wild individuals from India. While WGS provides a complete base-pair resolution of the genomic information, RADseq enables the genotyping of larger sample sets at a lower cost. Combining a subset of samples sequenced at medium depth with a larger dataset genotyped using RADseq is helpful for balancing genomic resolution with sample size. The integration of these two approaches allowed us to make broad population-level inference while at the same time retaining the ability to capture fine-scale genomic variations within a representative subset. This combined strategy can be applied to other endangered species where resources are limited but large sample sizes are needed, offering a cost-effective solution to obtain both fine-scale genomic resolution and population-wide insights, better supporting management decisions.

Our results demonstrate that genomic approaches can inform the impact of animal management on semi-captive populations, which are particularly vulnerable to inbreeding and reductions in effective population size, due to the impact of suboptimal human management. We highlight the need for genomic monitoring as a fundamental element of modern conservation, offering tools to assess the status of endangered populations and anticipate genetic erosion. Our work provides not only new insights into the conservation of Asian elephants but also a transferable framework for integrating genomic and demographic data to manage endangered species under human influence.

## Supplementary Information

Supplementary Material 1.

## Data Availability

The datasets generated and analysed during the current study are available in the NCBI repository, under BioProject ID PRJNA1359389.
