# Supplementary material for: Population genomic assessment of semi-captive Asian elephants (Elephas maximus) from Myanmar: endangered species management and conservation implications
Source: BMC Genomics. 2026 May 9;27:482. doi: 10.1186/s12864-026-12912-7 (PMC13185193; doi:10.1186/s12864-026-12912-7)
Supplement: Supplementary file 1 — Supplementary Material 1. [file 12864_2026_12912_MOESM1_ESM.docx]

## Supplementary

***Supplementary Table 1.*** *Summary of sequencing information for the individuals included in this study. For each sample it is reported the internal paper identifier, NCBI accession numbers for RADseq and WGS data, BioSample accession, mean WGS coverage, number of raw reads obtained from RADseq and WGS, and the sex of the individual.*

| **Paper ID** | **NCBI RADseq ID** | **NCBI WGS ID** | **Biosample accession** | **Mean coverage** | **RADseq raw reads** | **WGS raw reads** | **Sex** |
| --- | --- | --- | --- | --- | --- | --- | --- |
| 1 | Ele_RAD_013 | no WGS data | SAMN53163385 |  | 2.607.445 |  | F |
| 2 | Ele_RAD_014 | no WGS data | SAMN53163386 |  | 3.312.322 |  | F |
| 3 | Ele_RAD_015 | no WGS data | SAMN53163387 |  | 2.726.145 |  | F |
| 4 | Ele_RAD_016 | no WGS data | SAMN53163388 |  | 2.730.884 |  | F |
| 5 | Ele_RAD_017 | Ele_WGS_01 | SAMN53163389 | 21.44 | 3.322.880 | 340240793 | F |
| 6 | Ele_RAD_018 | no WGS data | SAMN53163390 |  | 2.891.501 |  | F |
| 7 | Ele_RAD_019 | Ele_WGS_02 | SAMN53163391 | 13.55 | 2.596.371 | 193221206 | M |
| 8 | Ele_RAD_020 | Ele_WGS_03 | SAMN53163392 | 20.16 | 2.664.061 | 299107478 | F |
| 9 | Ele_RAD_021 | no WGS data | SAMN53163393 |  | 3.411.607 |  | F |
| 10 | Ele_RAD_022 | no WGS data | SAMN53163394 |  | 2.278.838 |  | M |
| 11 | Ele_RAD_023 | Ele_WGS_05 | SAMN53163395 | 21.19 | 2.776.588 | 328104004 | F |
| 12 | Ele_RAD_024 | Ele_WGS_06 | SAMN53163396 | 15.32 | 2.669.234 | 234299312 | F |
| 13 | Ele_RAD_025 | no WGS data | SAMN53163397 |  | 2.628.314 |  | F |
| 14 | Ele_RAD_026 | Ele_WGS_07 | SAMN53163398 | 21.75 | 3.339.055 | 336569115 | F |
| 15 | Ele_RAD_027 | Ele_WGS_08 | SAMN53163399 | 15.76 | 2.293.549 | 246486440 | M |
| 16 | Ele_RAD_028 | Ele_WGS_09 | SAMN53163400 | 20.49 | 2.891.697 | 315211698 | F |
| 17 | Ele_RAD_029 | Ele_WGS_10 | SAMN53163401 | 19.91 | 3.239.057 | 305658638 | M |
| 18 | Ele_RAD_030 | Ele_WGS_11 | SAMN53163402 | 19.56 | 2.715.696 | 337271951 | F |
| 19 | Ele_RAD_031 | Ele_WGS_12 | SAMN53163403 | 19.56 | 3.508.144 | 304585206 | F |
| 20 | Ele_RAD_032 | Ele_WGS_13 | SAMN53163404 | 21.18 | 3.141.974 | 335025866 | F |
| 21 | Ele_RAD_033 | no WGS data | SAMN53163405 |  | 3.087.341 |  | M |
| 22 | Ele_RAD_034 | no WGS data | SAMN53163406 |  | 6.756.335 |  | F |
| 23 | Ele_RAD_035 | Ele_WGS_14 | SAMN53163407 | 21.12 | 3.177.448 | 305220729 | M |
| 24 | Ele_RAD_036 | no WGS data | SAMN53163408 |  | 6.770.329 |  | F |
| 25 | Ele_RAD_037 | no WGS data | SAMN53163409 |  | 3.030.070 |  | F |
| 26 | Ele_RAD_038 | Ele_WGS_15 | SAMN53163410 | 11.69 | 6.047.578 | 179381686 | F |
| 27 | Ele_RAD_039 | no WGS data | SAMN53163411 |  | 7.041.486 |  | F |
| 28 | Ele_RAD_040 | Ele_WGS_16 | SAMN53163412 | 18.94 | 3.750.012 | 269281745 | M |
| 29 | Ele_RAD_041 | no WGS data | SAMN53163413 |  | 3.214.367 |  | F |
| 30 | Ele_RAD_042 | no WGS data | SAMN53163414 |  | 3.624.425 |  | F |
| 31 | Ele_RAD_043 | Ele_WGS_17 | SAMN53163415 | 12.77 | 2.904.657 | 187766027 | M |
| 32 | Ele_RAD_044 | no WGS data | SAMN53163416 |  | 3.483.258 |  | F |
| 33 | Ele_RAD_045 | Ele_WGS_18 | SAMN53163417 | 10.67 | 2.673.133 | 164503282 | F |
| 34 | Ele_RAD_046 | Ele_WGS_19 | SAMN53163418 | 13.93 | 3.186.530 | 210228866 | F |
| 35 | Ele_RAD_047 | no WGS data | SAMN53163419 |  | 3.066.668 |  | F |
| 36 | Ele_RAD_048 | no WGS data | SAMN53163420 |  | 3.537.775 |  | F |
| 37 | Ele_RAD_049 | Ele_WGS_20 | SAMN53163421 | 21.89 | 1.644.747 | 337816378 | M |
| 38 | Ele_RAD_050 | Ele_WGS_21 | SAMN53163422 | 17.32 | 2.924.337 | 267109982 | F |
| 39 | Ele_RAD_051 | no WGS data | SAMN53163423 |  | 3.076.974 |  | F |
| 40 | Ele_RAD_052 | Ele_WGS_22 | SAMN53163424 | 10.68 | 2.667.579 | 164829049 | M |
| 41 | Ele_RAD_053 | Ele_WGS_23 | SAMN53163425 | 19.85 | 2.977.624 | 296400270 | M |
| 42 | Ele_RAD_054 | Ele_WGS_24 | SAMN53163426 | 12.85 | 3.196.123 | 185926905 | M |
| 43 | Ele_RAD_055 | no WGS data | SAMN53163427 |  | 3.022.554 |  | F |
| 44 | Ele_RAD_056 | no WGS data | SAMN53163428 |  | 2.830.513 |  | F |
| 45 | Ele_RAD_057 | no WGS data | SAMN53163429 |  | 3.131.442 |  | F |
| 46 | Ele_RAD_058 | Ele_WGS_25 | SAMN53163430 | 16.26 | 7.088.545 | 250198404 | F |
| 47 | Ele_RAD_059 | no WGS data | SAMN53163431 |  | 2.990.466 |  | F |
| 48 | Ele_RAD_060 | no WGS data | SAMN53163432 |  | 3.094.361 |  | F |
| 49 | Ele_RAD_061 | no WGS data | SAMN53163433 |  | 4.164.076 |  | F |
| 50 | Ele_RAD_062 | no WGS data | SAMN53163434 |  | 2.917.787 |  | F |
| 51 | Ele_RAD_063 | Ele_WGS_26 | SAMN53163435 | 22.71 | 2.917.271 | 339784071 | F |
| 52 | Ele_RAD_064 | Ele_WGS_27 | SAMN53163436 | 21.67 | 2.738.034 | 347621707 | M |
| 53 | Ele_RAD_065 | no WGS data | SAMN53163437 |  | 3.272.389 |  | F |
| 54 | Ele_RAD_066 | no WGS data | SAMN53163438 |  | 3.122.139 |  | F |
| 55 | Ele_RAD_067 | Ele_WGS_28 | SAMN53163439 | 22.37 | 3.438.269 | 346084425 | F |
| 56 | Ele_RAD_068 | no WGS data | SAMN53163440 |  | 3.469.032 |  | F |
| 57 | Ele_RAD_069 | Ele_WGS_29 | SAMN53163441 | 17.74 | 2.801.545 | 274219492 | F |
| 58 | Ele_RAD_070 | Ele_WGS_30 | SAMN53163442 | 20.25 | 2.997.804 | 309592939 | M |
| 59 | Ele_RAD_071 | no WGS data | SAMN53163443 |  | 2.506.119 |  | F |
| 60 | Ele_RAD_072 | no WGS data | SAMN53163444 |  | 3.319.599 |  | F |
| 61 | Ele_RAD_073 | Ele_WGS_31 | SAMN53163445 | 16.49 | 2.825.262 | 264787999 | F |
| 62 | Ele_RAD_074 | no WGS data | SAMN53163446 |  | 3.340.537 |  | F |
| 63 | Ele_RAD_075 | no WGS data | SAMN53163447 |  | 2.540.418 |  | F |
| 64 | Ele_RAD_076 | no WGS data | SAMN53163448 |  | 2.538.705 |  | F |
| 65 | Ele_RAD_077 | no WGS data | SAMN53163449 |  | 6.841.873 |  | F |
| 66 | Ele_RAD_078 | no WGS data | SAMN53163450 |  | 3.067.885 |  | F |
| 67 | Ele_RAD_079 | Ele_WGS_32 | SAMN53163451 | 10.68 | 2.960.464 | 162749487 | F |
| 68 | Ele_RAD_080 | Ele_WGS_33 | SAMN53163452 | 22.24 | 6.313.699 | 340936434 | F |
| 69 | Ele_RAD_081 | Ele_WGS_41 | SAMN53163453 | 19.43 | 2.548.472 | 304661834 | F |
| 70 | Ele_RAD_082 | no WGS data | SAMN53163454 |  | 3.306.819 |  | F |
| 71 | Ele_RAD_083 | Ele_WGS_34 | SAMN53163455 | 15.05 | 3.001.327 | 229438064 | F |
| 72 | Ele_RAD_084 | no WGS data | SAMN53163456 |  | 2.043.025 |  | M |
| 73 | Ele_RAD_085 | Ele_WGS_35 | SAMN53163457 | 18.16 | 3.209.455 | 271343898 | M |
| 74 | Ele_RAD_086 | no WGS data | SAMN53163458 |  | 5.912.243 |  | F |
| 75 | Ele_RAD_087 | Ele_WGS_36 | SAMN53163459 | 16.49 | 3.672.671 | 335409134 | F |
| 76 | Ele_RAD_088 | Ele_WGS_37 | SAMN53163460 | 20.97 | 2.561.806 | 297067615 | M |
| 77 | Ele_RAD_089 | Ele_WGS_38 | SAMN53163461 | 23.94 | 1.849.983 | 343380836 | M |
| 78 | Ele_RAD_090 | no WGS data | SAMN53163462 |  | 5.897.450 |  | F |
| 79 | Ele_RAD_091 | Ele_WGS_43 | SAMN53163463 | 16.30 | 3.154.279 | 245623529 | F |
| 80 | Ele_RAD_092 | Ele_WGS_39 | SAMN53163464 | 20.52 | 3.116.881 | 312170568 | F |
| 81 | Ele_RAD_093 | Ele_WGS_40 | SAMN53163465 | 20.11 | 5.095.752 | 317559238 | F |
| 82 | Ele_RAD_094 | Ele_WGS_42 | SAMN53163466 | 18.19 | 3.322.754 | 276632124 | M |
| 83 | Ele_RAD_095 | no WGS data | SAMN53163467 |  | 3.440.867 |  | F |
| 84 | Ele_RAD_096 | no WGS data | SAMN53163468 |  | 2.939.099 |  | M |
| 85 | Ele_RAD_097 | Ele_WGS_44 | SAMN53163469 | 18.45 | 3.034.091 | 287457869 | M |
| 86 | Ele_RAD_098 | no WGS data | SAMN53163470 |  | 2.437.308 |  | M |
| 87 | Ele_RAD_099 | no WGS data | SAMN53163471 |  | 2.619.623 |  | M |
| 88 | Ele_RAD_100 | no WGS data | SAMN53163472 |  | 3.290.318 |  | M |
| 89 | Ele_RAD_101 | no WGS data | SAMN53163473 |  | 2.761.457 |  | M |
| 90 | Ele_RAD_102 | no WGS data | SAMN53163474 |  | 2.912.939 |  | F |
| 91 | Ele_RAD_103 | no WGS data | SAMN53163475 |  | 3.544.660 |  | F |
| 92 | Ele_RAD_104 | no WGS data | SAMN53163476 |  | 3.334.480 |  | F |
| 93 | Ele_RAD_105 | no WGS data | SAMN53163477 |  | 2.644.027 |  | F |
| 94 | Ele_RAD_106 | Ele_WGS_47 | SAMN53163478 | 20.80 | 3.113.689 | 324884555 | F |
| 95 | Ele_RAD_107 | no WGS data | SAMN53163479 |  | 2.901.303 |  | F |
| 96 | Ele_RAD_108 | Ele_WGS_45 | SAMN53163480 | 22.61 | 3.182.403 | 330109264 | M |
| 97 | Ele_RAD_109 | Ele_WGS_46 | SAMN53163481 | 22.80 | 2.389.197 | 326314638 | M |
| 98 | Ele_RAD_110 | no WGS data | SAMN53163482 |  | 2.868.881 |  | M |
| 99 | Ele_RAD_111 | no WGS data | SAMN53163483 |  | 5.885.584 |  | F |
| 100 | Ele_RAD_112 | no WGS data | SAMN53163484 |  | 2.522.543 |  | F |
| 101 | Ele_RAD_113 | no WGS data | SAMN53163485 |  | 5.959.146 |  | F |
| 102 | Ele_RAD_114 | no WGS data | SAMN53163486 |  | 5.810.801 |  | F |
| 103 | Ele_RAD_115 | Ele_WGS_48 | SAMN53163487 | 18.21 | 2.469.344 | 270459436 | F |
| 104 | Ele_RAD_116 | no WGS data | SAMN53163488 |  | 4.538.059 |  | F |
| 105 | Ele_RAD_117 | no WGS data | SAMN53163489 |  | 3.057.639 |  | F |
| 106 | Ele_RAD_118 | no WGS data | SAMN53163490 |  | 3.636.812 |  | F |
| 107 | Ele_RAD_119 | no WGS data | SAMN53163491 |  | 2.709.657 |  | F |
| 108 | Ele_RAD_120 | Ele_WGS_49 | SAMN53163492 | 18.61 | 2.249.365 | 278696248 | F |
| 109 | Ele_RAD_121 | no WGS data | SAMN53163493 |  | 2.419.708 |  | F |
| 110 | Ele_RAD_122 | no WGS data | SAMN53163494 |  | 7.003.724 |  | F |
| 111 | Ele_RAD_123 | no WGS data | SAMN53163495 |  | 2.766.606 |  | M |
| 112 | Ele_RAD_124 | no WGS data | SAMN53163496 |  | 3.032.218 |  | M |
| 113 | Ele_RAD_125 | no WGS data | SAMN53163497 |  | 3.179.897 |  | F |
| 114 | Ele_RAD_126 | Ele_WGS_50 | SAMN53163498 | 23.09 | 3.009.373 | 331634281 | M |
| 115 | Ele_RAD_127 | no WGS data | SAMN53163499 |  | 2.740.337 |  | M |
| 116 | Ele_RAD_128 | no WGS data | SAMN53163500 |  | 3.361.351 |  | F |
| 117 | Ele_RAD_129 | no WGS data | SAMN53163501 |  | 2.734.219 |  | F |
| 118 | Ele_RAD_130 | no WGS data | SAMN53163502 |  | 3.177.411 |  | F |
| 119 | Ele_RAD_131 | no WGS data | SAMN53163503 |  | 2.497.079 |  | M |
| 120 | Ele_RAD_132 | Ele_WGS_51 | SAMN53163504 | 15.60 | 5.807.379 | 244510795 | F |
| 121 | Ele_RAD_133 | no WGS data | SAMN53163505 |  | 2.152.127 |  | F |
| 122 | Ele_RAD_134 | no WGS data | SAMN53163506 |  | 3.595.917 |  | M |
| 123 | Ele_RAD_135 | no WGS data | SAMN53163507 |  | 1.880.267 |  | F |
| 124 | Ele_RAD_136 | no WGS data | SAMN53163508 |  | 2.650.785 |  | F |
| 125 | Ele_RAD_137 | no WGS data | SAMN53163509 |  | 3.753.972 |  | F |
| 126 | Ele_RAD_138 | no WGS data | SAMN53163510 |  | 2.410.541 |  | F |
| 127 | Ele_RAD_139 | no WGS data | SAMN53163511 |  | 2.355.978 |  | F |
| 128 | Ele_RAD_140 | no WGS data | SAMN53163512 |  | 3.088.058 |  | F |
| 129 | Ele_RAD_141 | no WGS data | SAMN53163513 |  | 3.045.582 |  | F |
| 130 | Ele_RAD_142 | no WGS data | SAMN53163514 |  | 2.080.550 |  | M |
| 131 | Ele_RAD_143 | no WGS data | SAMN53163515 |  | 2.972.241 |  | M |
| 132 | Ele_RAD_144 | no WGS data | SAMN53163516 |  | 2.436.858 |  | F |
| 133 | Ele_RAD_145 | Ele_WGS_52 | SAMN53163517 | 8.53 | 3.747.649 | 270589246 | M |
| 134 | Ele_RAD_146 | no WGS data | SAMN53163518 |  | 2.080.266 |  | F |
| 135 | Ele_RAD_147 | no WGS data | SAMN53163519 |  | 2.955.965 |  | F |
| 136 | Ele_RAD_148 | no WGS data | SAMN53163520 |  | 3.440.125 |  | M |
| 137 | Ele_RAD_149 | no WGS data | SAMN53163521 |  | 2.919.718 |  | M |
| 138 | Ele_RAD_150 | no WGS data | SAMN53163522 |  | 2.726.040 |  | F |
| 139 | Ele_RAD_151 | no WGS data | SAMN53163523 |  | 1.733.332 |  | F |
| 140 | Ele_RAD_152 | no WGS data | SAMN53163524 |  | 2.435.122 |  | M |
| 142 | Ele_RAD_153 | Ele_WGS_54 | SAMN53163525 | 18.49 | 3.235.103 | 264861873 | M |
| 143 | Ele_RAD_154 | no WGS data | SAMN53163526 |  | 2.382.285 |  | F |
| 144 | Ele_RAD_155 | no WGS data | SAMN53163527 |  | 2.977.869 |  | F |
| 145 | Ele_RAD_156 | no WGS data | SAMN53163528 |  | 3.029.076 |  | F |
| 146 | Ele_RAD_157 | Ele_WGS_55 | SAMN53163529 | 21.33 | 3.183.199 | 310953144 | M |
| 147 | Ele_RAD_158 | no WGS data | SAMN53163530 |  | 2.402.342 |  | M |
| 148 | Ele_RAD_159 | no WGS data | SAMN53163531 |  | 3.399.906 |  | F |
| 149 | Ele_RAD_160 | no WGS data | SAMN53163532 |  | 3.356.860 |  | M |
| 150 | Ele_RAD_161 | no WGS data | SAMN53163533 |  | 3.718.692 |  | M |
| 151 | Ele_RAD_162 | no WGS data | SAMN53163534 |  | 2.955.307 |  | F |
| 152 | Ele_RAD_163 | no WGS data | SAMN53163535 |  | 2.766.339 |  | F |
| 153 | Ele_RAD_164 | no WGS data | SAMN53163536 |  | 3.036.522 |  | F |
| 154 | Ele_RAD_165 | no WGS data | SAMN53163537 |  | 3.456.047 |  | F |
| 155 | Ele_RAD_166 | no WGS data | SAMN53163538 |  | 2.316.863 |  | M |
| 156 | Ele_RAD_167 | no WGS data | SAMN53163539 |  | 3.219.550 |  | F |
| 157 | Ele_RAD_168 | no WGS data | SAMN53163540 |  | 3.058.508 |  | F |
| 158 | Ele_RAD_169 | no WGS data | SAMN53163541 |  | 1.501.335 |  | M |
| 159 | Ele_RAD_170 | no WGS data | SAMN53163542 |  | 3.429.371 |  | M |
| 160 | Ele_RAD_171 | no WGS data | SAMN53163543 |  | 3.731.252 |  | M |
| 161 | Ele_RAD_172 | no WGS data | SAMN53163544 |  | 2.918.303 |  | F |
| 162 | Ele_RAD_173 | Ele_WGS_56 | SAMN53163545 | 23.58 | 1.694.531 | 342450974 | M |
| 163 | Ele_RAD_174 | no WGS data | SAMN53163546 |  | 3.668.227 |  | F |
| 164 | Ele_RAD_175 | Ele_WGS_57 | SAMN53163547 | 21.68 | 3.005.316 | 354754005 | F |
| 165 | Ele_RAD_176 | no WGS data | SAMN53163548 |  | 2.921.221 |  | M |
| 166 | Ele_RAD_177 | no WGS data | SAMN53163549 |  | 3.466.321 |  | M |
| 167 | Ele_RAD_178 | Ele_WGS_58 | SAMN53163550 | 22.23 | 3.438.269 | 314502886 | M |
| 168 | Ele_RAD_179 | no WGS data | SAMN53163551 |  | 3.983.903 |  | M |
| 169 | Ele_RAD_180 | no WGS data | SAMN53163552 |  | 2.667.923 |  | M |
| 170 | Ele_RAD_181 | Ele_WGS_59 | SAMN53163553 | 17.59 | 3.654.129 | 263992440 | F |
| 171 | Ele_RAD_182 | Ele_WGS_60 | SAMN53163554 | 19.95 | 3.490.756 | 308189481 | F |
| 172 | Ele_RAD_183 | no WGS data | SAMN53163555 |  | 2.774.242 |  | F |
| 173 | Ele_RAD_184 | no WGS data | SAMN53163556 |  | 2.501.377 |  | F |
| 174 | Ele_RAD_185 | no WGS data | SAMN53163557 |  | 3.500.240 |  | F |
| 175 | Ele_RAD_186 | no WGS data | SAMN53163558 |  | 2.973.516 |  | F |
| 176 | Ele_RAD_187 | no WGS data | SAMN53163559 |  | 3.160.036 |  | M |
| 177 | Ele_RAD_188 | Ele_WGS_61 | SAMN53163560 | 22.58 | 3.263.538 | 322914901 | M |
| 178 | Ele_RAD_189 | no WGS data | SAMN53163561 |  | 1.849.404 |  | F |
| 179 | Ele_RAD_190 | no WGS data | SAMN53163562 |  | 2.760.714 |  | F |
| 180 | Ele_RAD_191 | no WGS data | SAMN53163563 |  | 3.446.981 |  | F |
| 181 | Ele_RAD_192 | no WGS data | SAMN53163564 |  | 2.059.939 |  | M |
| 182 | Ele_RAD_193 | no WGS data | SAMN53163565 |  | 3.879.268 |  | M |
| 183 | Ele_RAD_194 | no WGS data | SAMN53163566 |  | 3.068.433 |  | M |
| 184 | Ele_RAD_195 | no WGS data | SAMN53163567 |  | 2.831.370 |  | F |
| 185 | Ele_RAD_196 | no WGS data | SAMN53163568 |  | 2.801.628 |  | F |
| 186 | Ele_RAD_197 | no WGS data | SAMN53163569 |  | 2.926.715 |  | F |
| 187 | Ele_RAD_198 | no WGS data | SAMN53163570 |  | 2.998.610 |  | F |
| 188 | Ele_RAD_199 | Ele_WGS_62 | SAMN53163571 | 17.67 | 3.344.115 | 275848051 | F |
| 189 | Ele_RAD_200 | no WGS data | SAMN53163572 |  | 3.299.644 |  | M |
| 190 | Ele_RAD_201 | Ele_WGS_63 | SAMN53163573 | 20.76 | 3.218.087 | 302834903 | M |
| 191 | Ele_RAD_202 | no WGS data | SAMN53163574 |  | 3.181.243 |  | F |
| 192 | Ele_RAD_203 | no WGS data | SAMN53163575 |  | 2.851.563 |  | F |
| 193 | Ele_RAD_204 | no WGS data | SAMN53163576 |  | 3.461.792 |  | M |
| 194 | Ele_RAD_205 | no WGS data | SAMN53163577 |  | 3.107.022 |  | M |
| 195 | Ele_RAD_206 | no WGS data | SAMN53163578 |  | 2.085.090 |  | F |
| 196 | Ele_RAD_207 | Ele_WGS_64 | SAMN53163579 |  | 3.319.233 |  | F |
| 197 | Ele_RAD_208 | no WGS data | SAMN53163580 |  | 3.180.917 |  | M |
| 198 | Ele_RAD_209 | no WGS data | SAMN53163581 |  | 3.155.822 |  | M |
| 199 | Ele_RAD_210 | no WGS data | SAMN53163582 |  | 3.667.796 |  | M |
| 200 | Ele_RAD_211 | no WGS data | SAMN53163583 |  | 3.540.374 |  | M |
| 201 | Ele_RAD_212 | no WGS data | SAMN53163584 |  | 3.187.055 |  | M |
| 202 | Ele_RAD_213 | no WGS data | SAMN53163585 |  | 3.377.714 |  | F |
| 203 | Ele_RAD_214 | no WGS data | SAMN53163586 |  | 2.669.457 |  | F |
| 204 | Ele_RAD_215 | no WGS data | SAMN53163587 |  | 2.555.991 |  | F |
| 205 | Ele_RAD_216 | no WGS data | SAMN53163588 |  | 2.845.911 |  | M |
| 206 | Ele_RAD_217 | no WGS data | SAMN53163589 |  | 3.261.628 |  | M |
| 207 | Ele_RAD_218 | no WGS data | SAMN53163590 |  | 3.474.091 |  | F |
| 208 | Ele_RAD_219 | no WGS data | SAMN53163591 |  | 3.420.234 |  | M |
| 209 | Ele_RAD_220 | no WGS data | SAMN53163592 |  | 3.077.803 |  | M |
| 210 | Ele_RAD_221 | no WGS data | SAMN53163593 |  | 2.763.129 |  | M |
| 211 | Ele_RAD_222 | no WGS data | SAMN53163594 |  | 3.006.384 |  | M |
| 212 | Ele_RAD_223 | no WGS data | SAMN53163595 |  | 3.449.183 |  | F |
| 213 | Ele_RAD_224 | no WGS data | SAMN53163596 |  | 3.834.021 |  | M |
| 214 | Ele_RAD_225 | no WGS data | SAMN53163597 |  | 2.948.792 |  | M |
| 215 | Ele_RAD_226 | no WGS data | SAMN53163598 |  | 1.853.869 |  | M |
| 216 | Ele_RAD_227 | no WGS data | SAMN53163599 |  | 3.764.351 |  | F |
| 217 | Ele_RAD_228 | no WGS data | SAMN53163600 |  | 3.076.969 |  | F |
| 218 | Ele_RAD_229 | no WGS data | SAMN53163601 |  | 2.884.958 |  | F |
| 219 | Ele_RAD_230 | no WGS data | SAMN53163602 |  | 3.195.992 |  | F |
| 220 | Ele_RAD_231 | no WGS data | SAMN53163603 |  | 3.124.180 |  | F |
| 221 | Ele_RAD_232 | no WGS data | SAMN53163604 |  | 2.563.922 |  | F |
| 222 | Ele_RAD_233 | no WGS data | SAMN53163605 |  | 2.975.624 |  | M |
| 223 | Ele_RAD_234 | no WGS data | SAMN53163606 |  | 3.492.351 |  | M |
| 224 | Ele_RAD_235 | Ele_WGS_04 | SAMN53163607 | 18.60 | 3.225.973 | 282298515 | M |
| 225 | Ele_RAD_236 | no WGS data | SAMN53163608 |  | 3.277.972 |  | F |
| 226 | Ele_RAD_237 | no WGS data | SAMN53163609 |  | 3.298.391 |  | M |
| 227 | Ele_RAD_238 | no WGS data | SAMN53163610 |  | 3.342.169 |  | F |
| 228 | Ele_RAD_239 | no WGS data | SAMN53163611 |  | 2.816.397 |  | M |
| 229 | Ele_RAD_240 | no WGS data | SAMN53163612 |  | 3.062.502 |  | M |
| 230 | Ele_RAD_241 | no WGS data | SAMN53163613 |  | 2.835.388 |  | M |
| 231 | Ele_RAD_242 | no WGS data | SAMN53163614 |  | 3.353.739 |  | M |
| 232 | Ele_RAD_243 | no WGS data | SAMN53163615 |  | 3.282.494 |  | F |
| 233 | Ele_RAD_244 | no WGS data | SAMN53163616 |  | 2.988.624 |  | F |
| 234 | Ele_RAD_245 | no WGS data | SAMN53163617 |  | 2.961.594 |  | M |
| 235 | Ele_RAD_246 | no WGS data | SAMN53163618 |  | 2.949.779 |  | M |
| 236 | Ele_RAD_247 | no WGS data | SAMN53163619 |  | 2.949.595 |  | F |
| 237 | Ele_RAD_248 | no WGS data | SAMN53163620 |  | 2.562.256 |  | M |
| 238 | Ele_RAD_249 | no WGS data | SAMN53163621 |  | 3.634.858 |  | F |
| 239 | Ele_RAD_250 | no WGS data | SAMN53163622 |  | 3.208.389 |  | M |
| 240 | Ele_RAD_251 | no WGS data | SAMN53163623 |  | 3.569.534 |  | F |
| 241 | Ele_RAD_252 | no WGS data | SAMN53163624 |  |  |  | M |
| 242 | Ele_RAD_253 | no WGS data | SAMN53163625 |  |  |  | M |
| 243 | Ele_RAD_254 | no WGS data | SAMN53163626 |  |  |  | M |
| 244 | Ele_RAD_255 | no WGS data | SAMN53163627 |  | 3.294.828 |  | NN |
| 245 | Ele_RAD_256 | no WGS data | SAMN53163628 |  |  |  | M |
| 246 | Ele_RAD_257 | no WGS data | SAMN53163629 |  |  |  | M |
| 247 | Ele_RAD_258 | no WGS data | SAMN53163630 |  |  |  | M |
| 248 | Ele_RAD_259 | no WGS data | SAMN53163631 |  |  |  | M |
| 249 | Ele_RAD_260 | no WGS data | SAMN53163632 |  |  |  | M |
| 250 | Ele_RAD_261 | no WGS data | SAMN53163633 |  |  |  | M |
| 251 | Ele_RAD_012 | no WGS data | SAMN53163384 |  | 2.813.413 |  | M |
| 252 | Ele_RAD_001 | no WGS data | SAMN53163373 |  | 3.711.232 |  | F |
| 253 | Ele_RAD_003 | no WGS data | SAMN53163375 |  | 2.581.874 |  | F |
| 254 | Ele_RAD_004 | no WGS data | SAMN53163376 |  | 2.921.110 |  | F |
| 255 | Ele_RAD_005 | no WGS data | SAMN53163377 |  | 3.524.037 |  | F |
| 256 | Ele_RAD_006 | no WGS data | SAMN53163378 |  | 2.771.927 |  | F |
| 257 | Ele_RAD_007 | no WGS data | SAMN53163379 |  | 2.624.244 |  | F |
| 258 | Ele_RAD_008 | no WGS data | SAMN53163380 |  | 2.877.342 |  | F |
| 259 | Ele_RAD_009 | no WGS data | SAMN53163381 |  | 2.887.241 |  | F |
| 260 | Ele_RAD_010 | no WGS data | SAMN53163382 |  | 3.514.147 |  | F |
| 262 | Ele_RAD_011 | no WGS data | SAMN53163383 |  | 3.452.266 |  | NN |

***Supplementary Table 2.*** *MTE camp name, camp acronym and district, total number of samples (N), number of samples born or captured in the camp area, observed heterozygosity and inbreeding (calculated only for camps with more than 10 samples).*

| **Camp** | **Acronym** | **District** | **N** | **N Captive** | **N Wild** | **H_o_** | **Inbreeding** |
| --- | --- | --- | --- | --- | --- | --- | --- |
| Bamauk | Bmk | Katha | 1 | 1 | 0 | - | - |
| Bhamo | Bmo | Bhamo | 1 | 1 | 0 | - | - |
| East Katha | EKa | Katha | 51 | 42 | 9 | 0.1871 | 0.0449 |
| East Mawlaik | EMa | Mawlaik | 3 | 3 | 0 | - | - |
| Homemalin | Hom | Hkamti | 4 | 2 | 2 |  | - |
| Htamanthi | Hta | Hkamti | 1 | 0 | 1 | - | - |
| Kawlin | Kaw | Kawlin | 129 | 114 | 15 | 0.1896 | 0.0436 |
| Mansi | Mns | Bhamo | 2 | 0 | 2 | - | - |
| Mawlaik | Mwl | Mawlaik | 1 | 0 | 1 | - | - |
| Moe Kaung | Mok | Mohnyin | 3 | 0 | 3 | - | - |
| Moemeik | Mem | Mongmit | 3 | 2 | 1 | - | - |
| Myitkyina | Myt | Myitkyina | 7 | 0 | 7 | - | - |
| Pyinmana | Pyn | Det Khi Na | 1 | 1 | 0 | - | - |
| Shwebo | Swb | Shwebo | 6 | 4 | 2 | - | - |
| Shweli | Swl | Mongmit | 1 | 1 | 0 | - | - |
| Taunggyi | Tgg | Taunggyi | 1 | 0 | 1 | - | - |
| Thandaung Gyi Stockade | Tnd | Hpa-An | 1 | 0 | 1 | - | - |
| Tharyarwady | Try | Thayarwady | 1 | 0 | 1 | - | - |
| West Katha | WKa | Katha | 38 | 31 | 7 | 0.1902 | 0.0446 |
| West Mawlaik | WMa | Mawlaik | 2 | 2 | 0 | - | - |

*
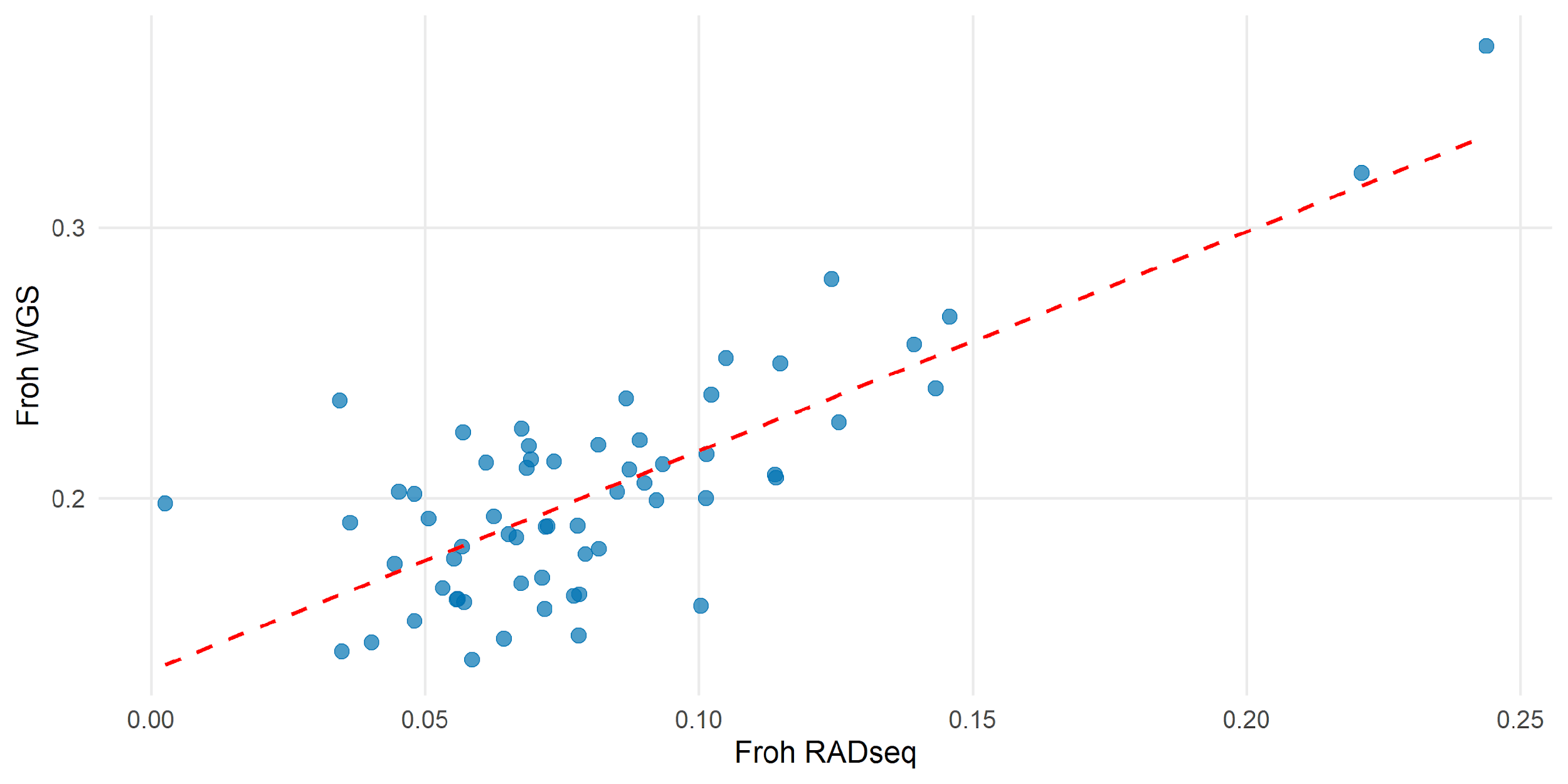
*

***Supplementary Figure 1.*** Relationship between F_ROH_ estimated from RADseq and WGS data. Each point represents an individual’s F_ROH_ value estimated using both datasets.

*
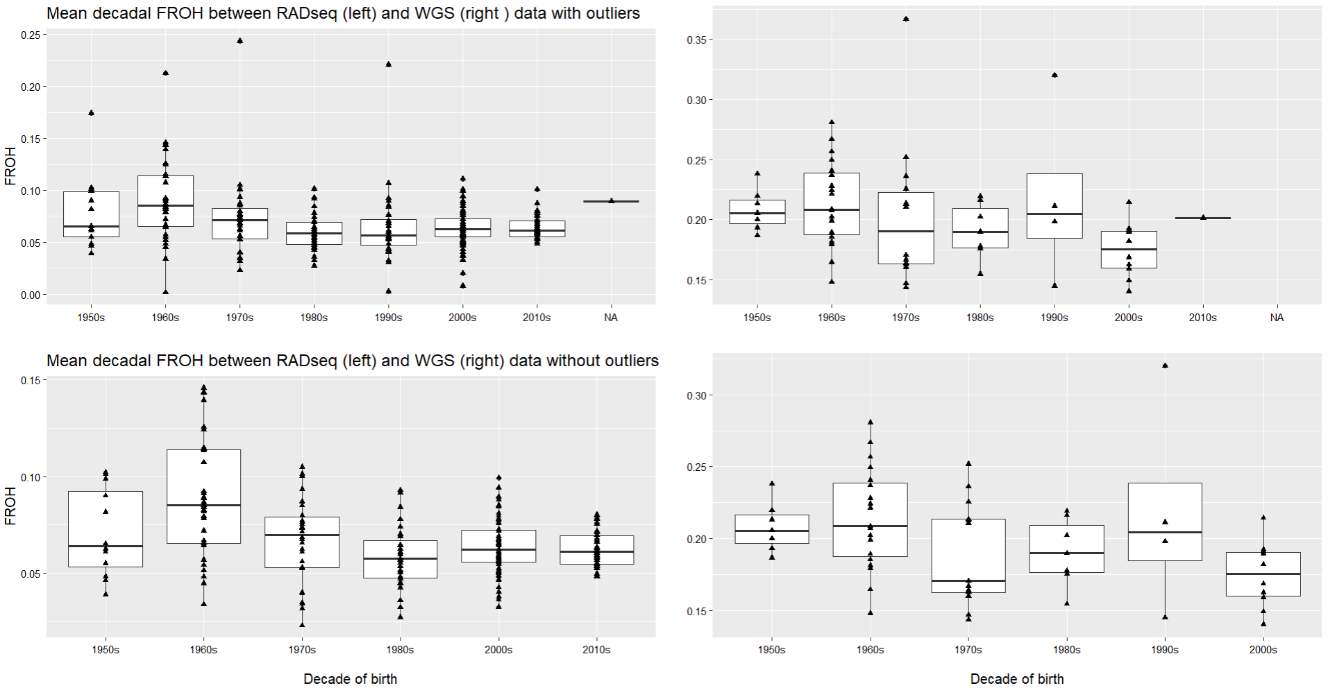
*

***Supplementary Figure 2.*** *Average values of F_ROH_ for individuals grouped by decade of birth.*


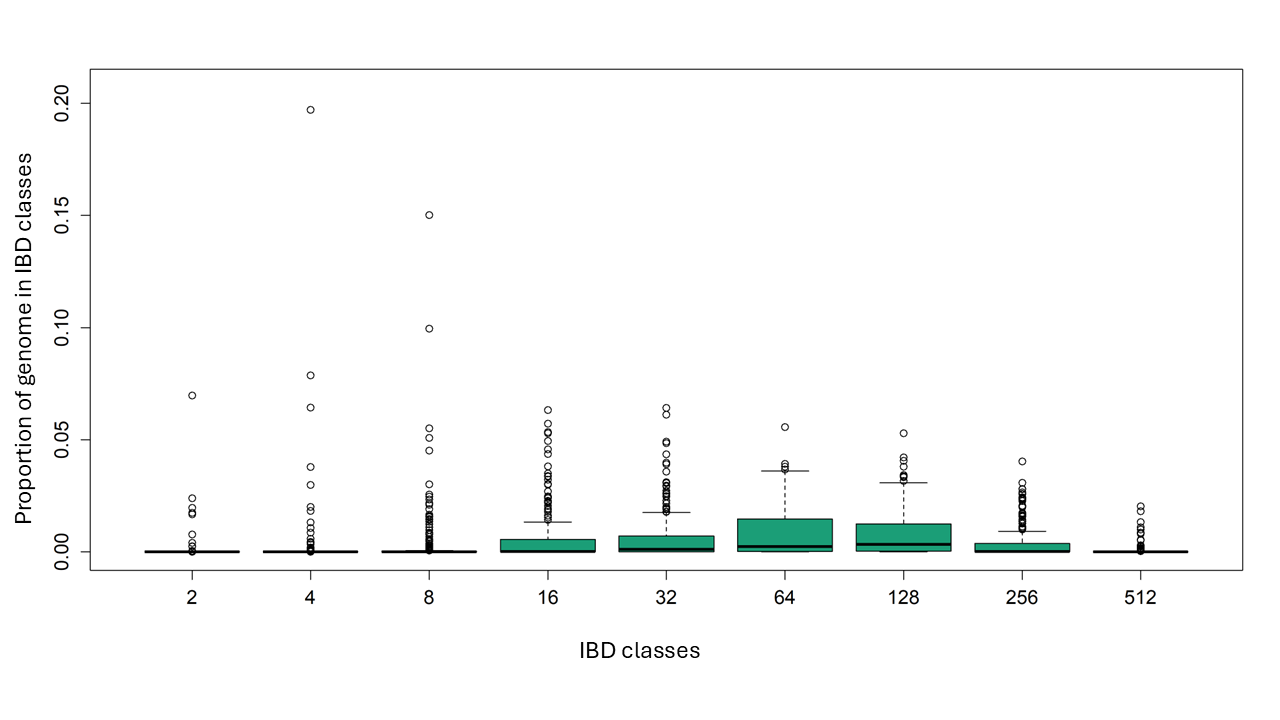


***Supplementary Figure 3.*** *Proportion of the genome assigned to different inbreeding classes for the RADseq dataset. Each box represents the distribution of the genomic proportion attributed to a specific class across individuals.*

*
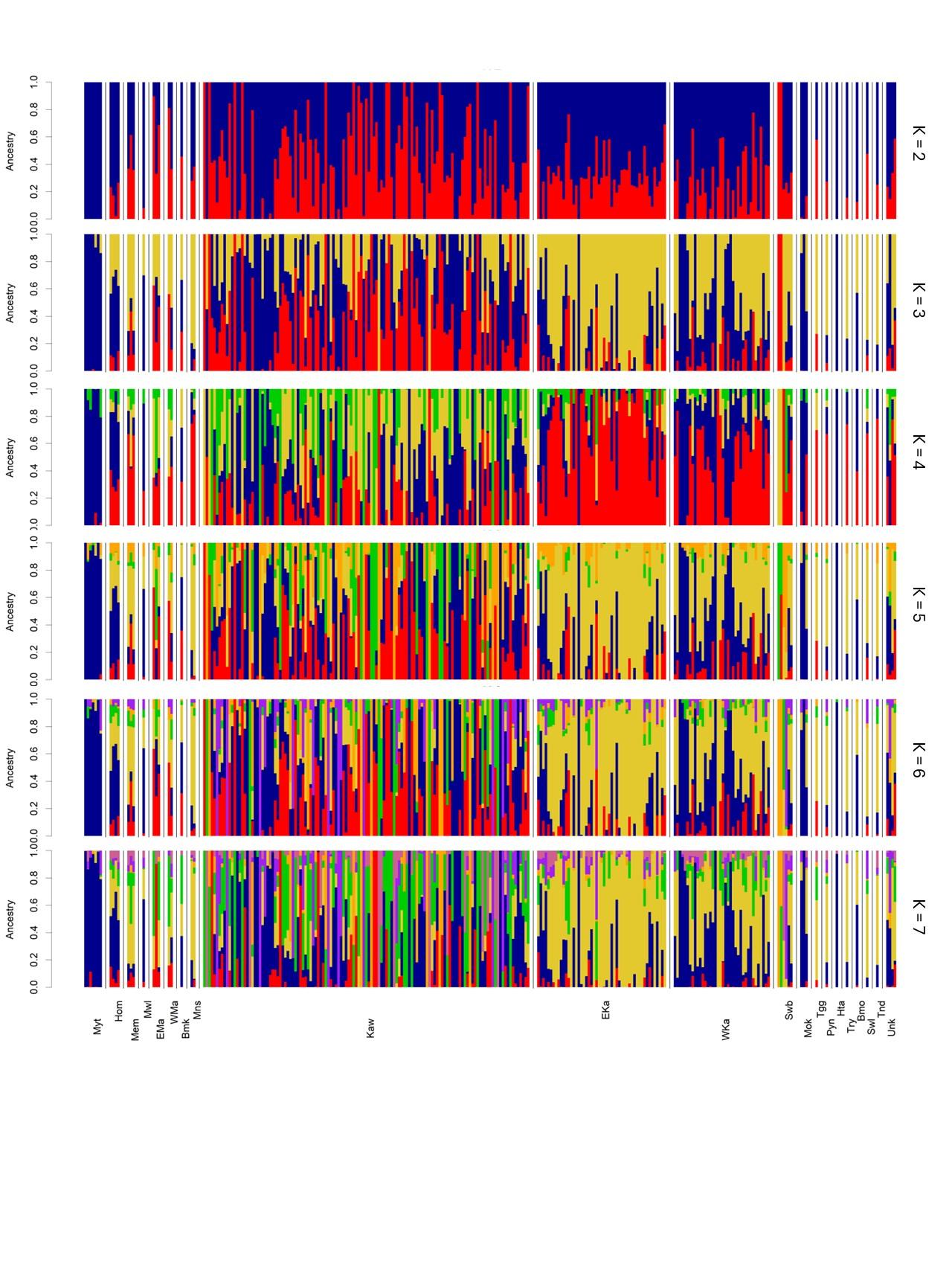
*

***Supplementary Figure 4.*** *Admixture plot for K 2 to 7 (K) for all samples clustered according to the camp of origin and ordered on a north to south geographical gradient. Camp acronym can be found in Supplementary table 2.*


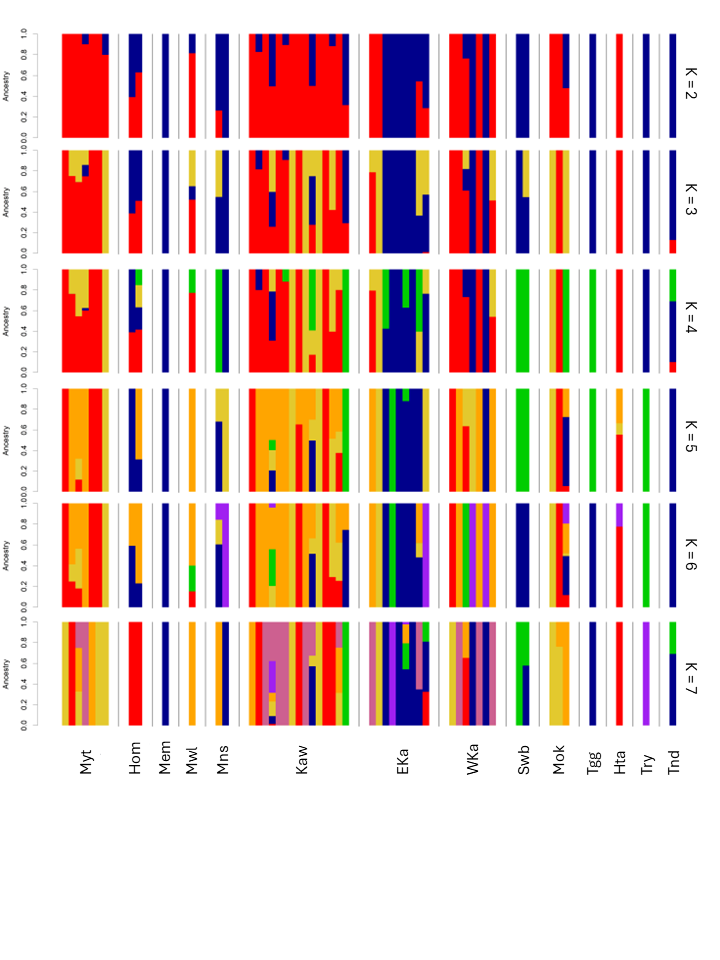


***Supplementary Figure 5.*** *Admixture plot for K from 2 to 7 of only wild born samples clustered according to the capture location and ordered on a north to south geographical gradient. Camp acronym can be found in Supplementary Table 1.*


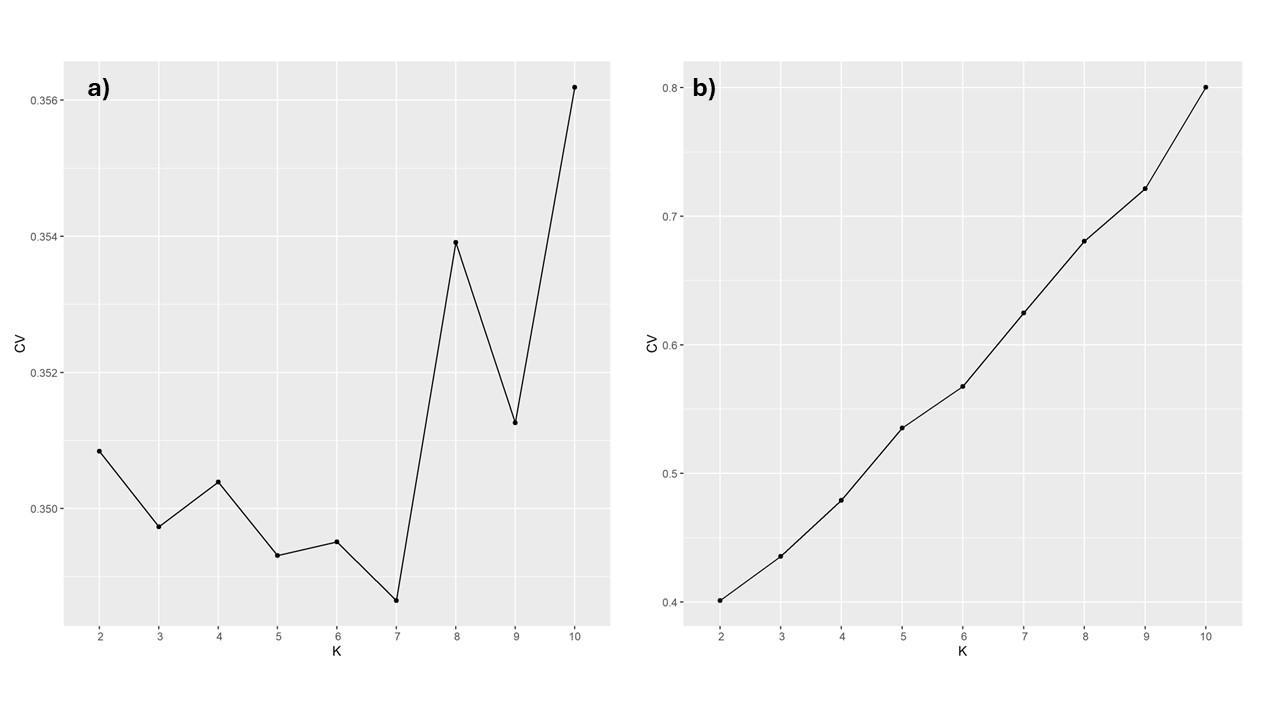


***Supplementary Figure 6a and b****: CV values for a) admixture analysis on all samples and b) only wild samples*


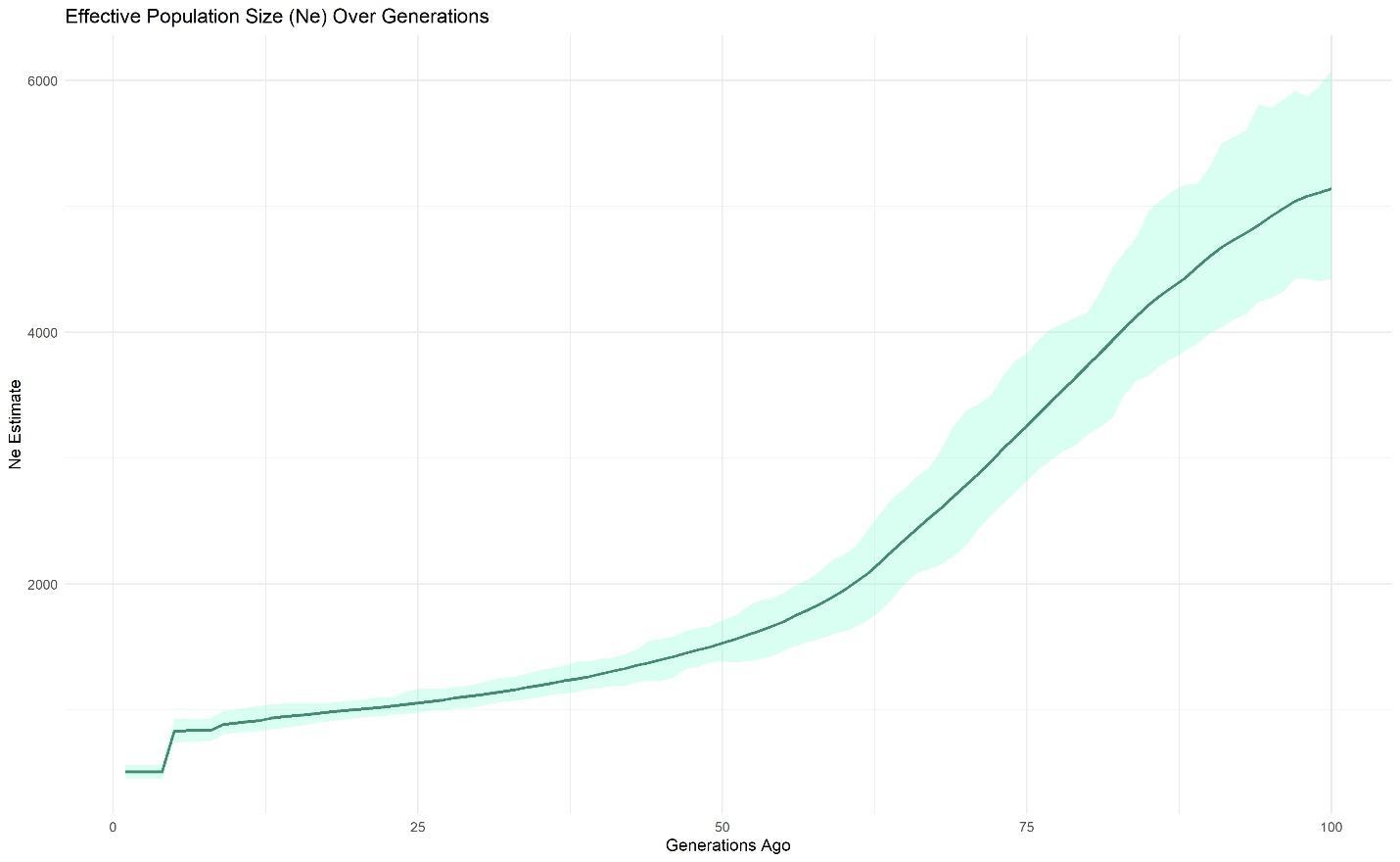


***Supplementary Figure 7.*** *Historical estimates of effective population size (Ne) over the past 100 generations, inferred using the RADseq dataset with the GONe software over 100 replicates on the full set of individuals. The solid line represents the median Ne estimate, while the shaded area indicates 95% confidence intervals.*

***Supplementary Table 3.*** *Distribution of SNPs per chromosome for each one of the RIM panels*

| ***Chromosome*** | ***N of SNPs x chromosome*** | | |
| --- | --- | --- | --- |
|  | *719 SNPs RIM panel* | *516 SNPs RIM panel* | *274 SNPs RIM panel* |
| *1* | *61* | *39* | *23* |
| *2* | *52* | *42* | *22* |
| *3* | *44* | *25* | *17* |
| *4* | *59* | *36* | *14* |
| *5* | *44* | *32* | *13* |
| *6* | *42* | *27* | *16* |
| *7* | *34* | *25* | *11* |
| *8* | *34* | *20* | *13* |
| *9* | *33* | *18* | *6* |
| *10* | *32* | *17* | *9* |
| *11* | *38* | *26* | *18* |
| *12* | *31* | *17* | *8* |
| *13* | *31* | *21* | *10* |
| *14* | *26* | *19* | *9* |
| *15* | *18* | *12* | *5* |
| *16* | *24* | *19* | *10* |
| *17* | *25* | *17* | *8* |
| *18* | *20* | *8* | *4* |
| *19* | *13* | *10* | *6* |
| *20* | *19* | *14* | *9* |
| *21* | *24* | *13* | *7* |
| *22* | *14* | *11* | *7* |
| *23* | *21* | *16* | *7* |
| *24* | *13* | *8* | *6* |
| *25* | *14* | *9* | *6* |
| *26* | *16* | *11* | *7* |
| *27* | *9* | *4* | *3* |
